# Landscape genetics and the genetic legacy of Upper Paleolithic and Mesolithic hunter-gatherers in the modern Caucasus

**DOI:** 10.1038/s41598-021-97519-6

**Published:** 2021-09-09

**Authors:** Alexander Gavashelishvili, Alexey Yanchukov, David Tarkhnishvili, Marine Murtskhvaladze, Irakli Akhvlediani, Ceren Kazancı

**Affiliations:** 1 Center of Biodiversity Studies, Institute of Ecology, Ilia State University, Cholokashvili Str. 5, 0162 Tbilisi, Georgia; 2 Department of Biology, Faculty of Arts and Sciences, Bülent Ecevit University, Zonguldak, Turkey; 3 School of Natural Sciences and Engineering, Ilia State University, Tbilisi, Georgia; 4 L. Sakvarelidze National Center for Disease Control and Public Health, Tbilisi, Georgia; 5 Georgian FTDNA Project, Calle Francisco Tierra N2, 9A., 48903 Barakalado, Vizcaya Spain; 6 Life Sciences, Institute of Ecology, Ilia State University, Cholokashvili Str. 5, 0162 Tbilisi, Georgia

**Keywords:** Computational biology and bioinformatics, Ecology, Genetics, Molecular biology, Ecology

## Abstract

This study clarifies the role of refugia and landscape permeability in the formation of the current genetic structure of peoples of the Caucasus. We report novel genome-wide data for modern individuals from the Caucasus, and analyze them together with available Paleolithic and Mesolithic individuals from Eurasia and Africa in order (1) to link the current and ancient genetic structures via landscape permeability, and (2) thus to identify movement paths between the ancient refugial populations and the Caucasus. The ancient genetic ancestry is best explained by landscape permeability implying that human movement is impeded by terrain ruggedness, swamps, glaciers and desert. Major refugial source populations for the modern Caucasus are those of the Caucasus, Anatolia, the Balkans and Siberia. In Rugged areas new genetic signatures take a long time to form, but once they do so, they remain for a long time. These areas act as time capsules harboring genetic signatures of ancient source populations and making it possible to help reconstruct human history based on patterns of variation today.

## Introduction

The Caucasus is a mountainous region located on the dividing line between Europe and Asia, between the Black and Caspian seas. In spite of relatively small geographic extent and mostly temperate climate, the diversity of natural landscapes, plant and animal species, and cultivars is unusually high in the Caucasus^1^. Thanks to this diversity the Caucasus is one of the global biodiversity hotspots that also contain considerable linguistic diversity, accounting for most of all languages on Earth^2,3^. The Caucasus provided some of the globe’s important refugia, where most of the terrestrial plants and animals (incl. humans) survived during a series of glacial maxima, and their current distribution largely reflects post-glacial expansion from these refugia^1^. These glacial refugia and the barriers to migration played an important role in human evolution, and generated much of the human genetic and ethno-linguistic patterns found in the world today^4–7^. The Caucasus contributed about half of the genetic ancestry of Yamnaya^8^—that is, pastoralists of the Pontic-Caspian steppe, who date from the Late Copper Age to the Early Bronze Age and made profound demographic and cultural impacts on much of Eurasia e.g. by spreading genomes, Indo-European languages and horse-riding^9–14^. It is hypothesized that Proto-Indo-European was generated from admixture between archaic languages from the Caucasus and Proto-Uralic languages in the Pontic-Caspian steppe^15,16^. Thus, the Caucasus has played an important role in the formation of past and present Eurasian genetic and cultural diversity.

As research technologies advance and more samples are obtained, studies have been unveiling more of the genetic variation of Caucasian populations in relation to geography and various indicators of ethnicity^7,17–24^. Autosomal genome and mtDNA variations in the Caucasus appear relatively homogenous, while the Y-chromosome diversity clearly shows geographic heterogeneity distinguishing the eastern from western Caucasians^7,17,24^. This east–west cline in the Caucasus was also shown for autosomal genome variation when Georgians were divided into sub-ethnic groups^22^. The studies of genome-wide autosomal profiles as well as the mitochondrial and Y chromosomal haplogroups support that there is genetic continuity in the present-day southern Caucasus stretching back at least 13,000 years to the Late Upper Palaeolithic^8,25^. In the North Caucasus this continuity was disrupted due to post-bronze age admixture with populations from the Eurasian Steppe^26^. At present genetic differentiation between ethnic/subethnic groups in the Caucasus correlates with landscape permeability to human movement—as determined by terrain ruggedness, forest cover, and snow cover—rather than ethnic or linguistic boundaries^22^.

Tarkhnishvili et al.^22^ hypothesized that the genetic makeup of the modern Caucasians was largely shaped up by human dispersal from few distinct glacial refugia in the last glacial period and the early Holocene, followed by less gene flow between the populations of the Greater Caucasus than between those of the rest of the Caucasus, where the populations have undergone substantial admixture in historical time. Understanding the nature of human movement from refugial populations was the primary motivation behind this study. Here we measure genome-wide genetic affinity between modern populations of the Caucasus and ancient populations of hunter-gatherers, test whether the genetic affinity between these populations is determined by geographic features, and infer major dispersal paths from refugial populations into the Caucasus. The results of this study allow us to reconstruct an integral picture of the peopling of the Caucasus from the last glacial period through the early Holocene.

## Methods

### Sampling and genotyping

**Figure 1:**
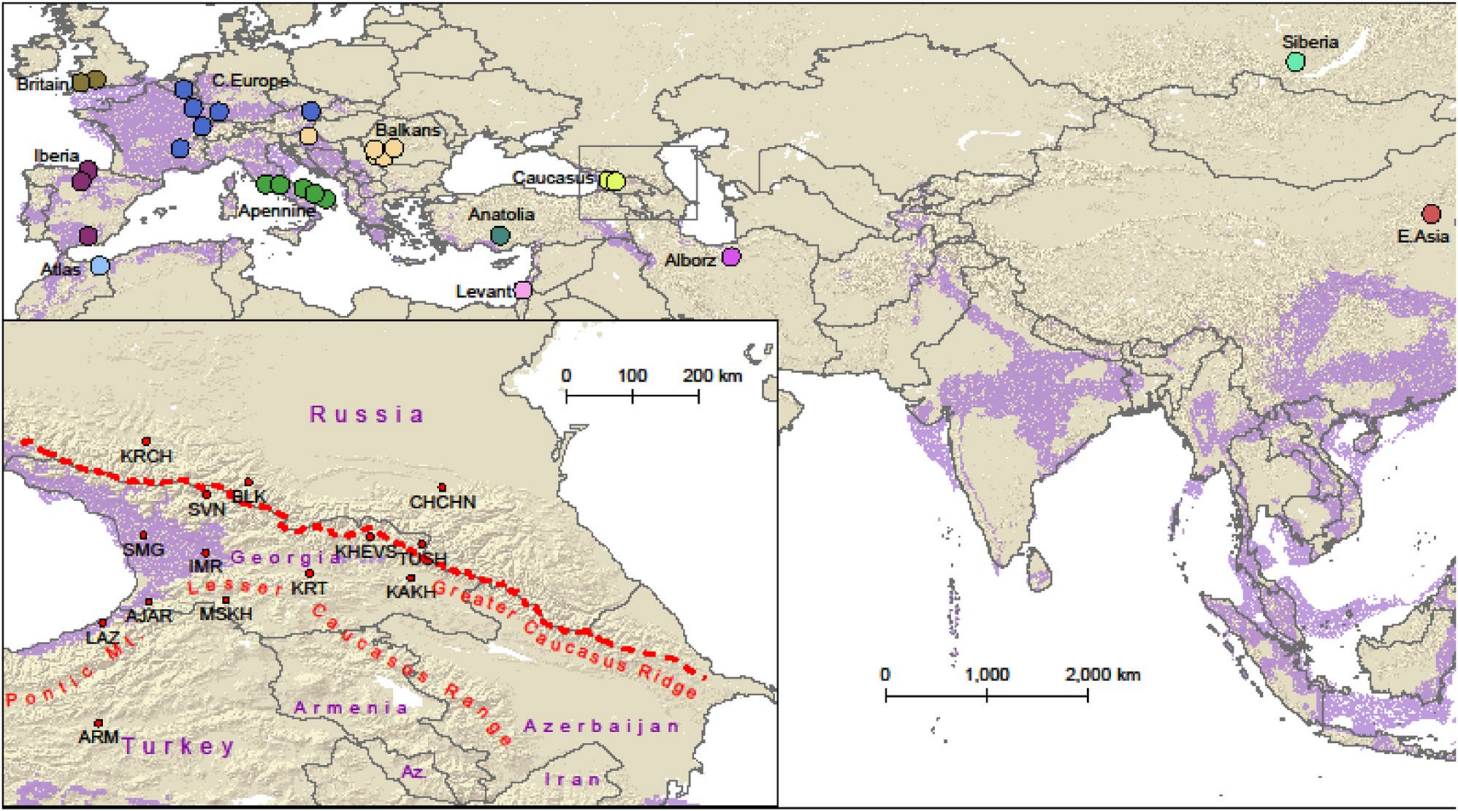
The distribution of the study populations: averaged centroids of ancient populations (uniquely colored points in the main map, see Table 2 for details) and hubs of the modern Caucasian populations (identified in the inset map, see Table 1 for details). Glacial human refugia extracted from Gavashelishvili and Tarkhnishvili^5^ are shaded in purple. The map is generated using QGIS Desktop 3.10.6-A Coruña (https://qgis.org).

We collected hair and cheek swab samples from 77 men from geographically and linguistically distinct groups of the Caucasus: Kartvelian speakers from Georgia and Turkey, Northeast Caucasian speakers and Turkic speakers from the Russian Federation and Armenian speakers from Georgia’s southern province of Javakheti, descendants of the families displaced from Mush and Erzurum provinces of eastern Turkey in the early nineteenth century (Table 1, Fig. 1). To maximize the representativeness of the genetic signature of each population, the samples were collected from locals with no ancestors from outside of the respective ethnic/geographic population over the last three generations. DNA was extracted from follicles of 10–12 male chest hairs and cheek swab samples. Extraction was performed using Qiagen DNeasy Blood and Tissue kit, following the manufacturer’s recommendations (Qiagen, Valencia, CA, USA). The DNA samples were genotyped for 693,719 autosomal and 17,678 X-chromosomal SNPs by Family Tree DNA (FTDNA—Gene By Gene, Ltd, Houston, TX, www.familytreedna.com).

**Table 1:** Modern study populations of the Caucasus. Latitude and longitude georeference population hubs.

Abbreviation of population name	Sample size	Population hub	Country	Ethnic/subethnic group	Longitude	Latitude	Linguistic family
AJAR	3	Keda	Georgia	Georgian/Adjara	41.94151	41.59755	Kartvelian
ARM	6	Erzurum	Turkey	Armenian	41.26181	39.95971	Indo-European
BLK	4	Bezengi	Russia	Balkar	43.28644	43.21567	Turkic
CHCHN	6	Shali	Russia	Chechen	45.90188	43.14471	Northeast Caucasian
IMR	5	Kutaisi	Georgia	Georgian/Imereti	42.70703	42.26331	Kartvelian
KAKH	5	Telavi	Georgia	Georgian/Kakheti	45.47792	41.92137	Kartvelian
KHEVS	4	Barisakho	Georgia	Georgian/Khevsureti	44.9235	42.47339	Kartvelian
KRCH	3	Karachayevsk	Russia	Karachay	41.9096	43.76891	Turkic
KRT	6	Gori	Georgia	Georgian/Kartli	44.10855	41.98558	Kartvelian
LAZ	13	Arhavi	Turkey	Laz	41.31929	41.31664	Kartvelian
MSKH	5	Akhaltsikhe	Georgia	Georgian/Meskheti	42.98199	41.62398	Kartvelian
SMG	5	Zugdidi	Georgia	Georgian/Samegrelo	41.86624	42.49668	Kartvelian
SVN	7	Mestia	Georgia	Georgian/Svaneti	42.72358	43.04344	Kartvelian
TUSH	5	Omalo	Georgia	Georgian/Tusheti	45.63018	42.37898	Kartvelian

Our dataset of modern Caucasian genotypes was supplemented with published 10 modern Mbuti (Supplementary Table S1) and 122 Upper Paleolithic-Mesolithic human genotypes, retrieved as a part of 1240 K dataset from David Reich’s Lab website, Harvard University (https://reich.hms.harvard.edu/downloadable-genotypes-present-day-and-ancient-dna-data-compiled-published-papers; see Supplementary Table S2 for details). The ancient genotypes were selected such that they either dated from the LGM or fell within the glacial refugia identified by Gavashelishvili and Tarkhnishvili^5^. We did so in order to maximize the genetic signature of potential refugial populations in our analysis. We divided the ancient genotypes into 2000-year-long intervals, and then grouped each of these intervals into geographic units (hereafter ancient populations, Table 2, Fig. 1). The modern and ancient genotypes were merged using PLINK 1.9 (PLINK 1.9: www.cog-genomics.org/plink/1.9/^27^.

**Table 2:** Ancient study populations. The ancient genotypes are divided into 2000-year-long intervals, and then each of these intervals is grouped into geographic units (i.e. ancient populations). Age, latitude and longitude are averaged across each ancient population (see Supplementary Table S2 for details).

ID	Ancient population	Sample size	Mean years before present (BP)	2 k Interval BP	Latitude	Longitude
Alborz16	Alborz	2	11,375	9950–11,950	35.5905	53.5005
Anatolia14	Anatolia	1	15,405	13,950–15,950	37.48333	33.03333
Apennine14	Apennine	2	14,127.5	13,950–15,950	42.04167	12.27167
Apennine16	Apennine	3	11,148.333	9950–11,950	41.96	13.54
Apennine17	Apennine	1	9125	7950–9950	41.96	13.54
Apennine5	Apennine	1	32,895	31,950–33,950	41.65	15.61
Apennine7	Apennine	1	28,975	27,950–29,950	40.73	17.57
Apennine8	Apennine	2	27,685	25,950–27,950	41.19	16.59
Atlas14	Atlas	7	14,490.286	13,950–15,950	34.80781	− 2.41089
Balkans1	Balkans	1	41,950	39,950–41,950	46.29917	16.07056
Balkans16	Balkans	10	10,599	9950–11,950	44.58344	22.01852
Balkans17	Balkans	33	8742.182	7950–9950	44.52966	22.09219
Balkans18	Balkans	5	7811.8	5950–7950	44.44482	22.7108
Balkans2	Balkans	1	39,610	37,950–39,950	45.12	21.9
Balkans5	Balkans	2	32,867.5	31,950–33,950	45.23	23.65
Britain16	Britain	3	10,418	9950–11,950	51.31036	− 2.75741
Britain17	Britain	2	9359	7950–9950	51.05035	− 4.09799
C_Europe14	C_Europe	6	14,944.333	13,950–15,950	48.446	8.136333
C_Europe15	C_Europe	2	13,312.5	11,950–13,950	47.155	6.66
C_Europe16	C_Europe	4	11,315	9950–11,950	45.195	4.7025
C_Europe17	C_Europe	3	9050	7950–9950	48.79667	5.85
C_Europe4	C_Europe	1	34,795	33,950–35,950	50.446	5.008
C_Europe6	C_Europe	7	30,548.571	29,950—31,950	48.51286	16.27571
C_Europe7	C_Europe	1	27,975	27,950–29,950	50.446	5.008
C_Europe8	C_Europe	2	26,917.5	25,950–27,950	50.446	5.008
Caucasus15	Caucasus	1	13,255	11,950–13,950	42.38	42.59
Caucasus17	Caucasus	1	9720	7950–9950	42.28	43.28
E_Asia2	E_Asia	1	39,475	37,950–39,950	39.39	115.52
Iberia12	Iberia	1	18,720	17,950–19,950	43.26	− 3.45
Iberia18	Iberia	4	7828.25	5950–7950	42.2878	− 4.06254
Iberia9	Iberia	1	24,450	23,950–25,950	37.4488	− 3.4297
Levant15	Levant	6	12,835.833	11,950–13,950	32.65	35.067
Siberia9	Siberia	1	24,305	23,950–25,950	52.9	103.5

### Ethics statement

The research team members, through their contacts in the studied communities, inquired whether locals would voluntarily participate in genetic research that would help clarify the genetic makeup of the Caucasus. A verbal agreement was made with volunteer donors of DNA samples, according to which the results would be communicated, electronically or in hard copy, with participants individually. Participants were informed that, upon the completion of the lab work, the research would be published without mentioning the names of sample donors. Those who agreed provided us with the envelopes containing their chest hairs or cheek swab samples, with the birthplace of their ancestors (last three generations) written on the envelope or a piece of paper. In accordance with the preferences of the sample donors, the agreement was verbal and not written. The envelopes and papers are stored as evidence of voluntary provision of the samples and the related information. Analysis of data was done anonymously, using only location and ethnic information; only the first and third authors of the manuscript had access to names associated with the samples. Therefore, this study was based on noninvasive and nonintrusive sampling (volunteers provided hair and swab samples they collected themselves), and the information destined for open publication does not contain any personal information. The study methodology and the procedure of verbal consent was discussed in detail with and approved by the members of the Ilia State University Commission for Ethical Issues before the field survey started, and the commission decided that formal ethical approval was not needed for conducting this study. This is confirmed in a letter from the commission chairman, a copy of which has been provided to the journal editor as part of the submission process.

### Genetic affinity and geography

First, we measured genetic affinity between the modern Caucasian populations, and between the modern populations and the ancient populations of hunter-gatherers, and then tested whether the genetic affinity between these populations was determined by geographic features. Data were mapped using QGIS Desktop 3.10.6-A Coruña, whereas graphs were created using the “ggplot2” package^28^ in R version 3.5.2^29^.

To evaluate genetic affinities and structure of the modern populations, we used Wright’s fixation index (Fst), inbreeding coefficient, admixture analysis and the principal component analysis (PCA). For these procedures we filtered the raw SNP genotypes in PLINK 1.9, first removing all SNPs with the minor allele frequency < 0.05, followed by LD pruning of markers that exceeded the pairwise correlation threshold of r^2^ > 0.3, calculated in windows of 50 bp size and 10 bp steps (–maf 0.05 –indep-pairwise 50 10 0.3). Since all individuals in our dataset possess a single copy of the X-chromosome, we did not expect any differential ploidy bias, and SNPs on the X were treated similarly to those on the autosomes. Fst pairwise values were calculated using the smartpca program of EIGENSOFT^30^ with default parameters, inbreed: YES, and fstonly: YES. The relationship between the modern populations based on Fst values was visualized by constructing a neighbor-joining tree using the “ape” package^31^ in R version 3.5.2. The average and standard deviation of the inbreeding coefficient for each population was calculated using “fhat2” estimate of PLINK 1.9. The LD pruned genotypes were used in ADMIXTURE 1.3.0^32^, performed in unsupervised mode in order to infer the population structure from the modern individuals. The number of clusters (k) was varied from 2 to 7 and the fivefold cross-validation error was calculated for each k^33^. We conducted principal components analysis in the smartpca program of EIGENSOFT^30^, using default parameters and the lsqproject: YES and numoutlieriter: 0 options. Eigenvectors of principal components were inferred with the modern populations from the Caucasus, while the ancient populations were then projected onto the PCA plots. We also assessed the relatedness between sampled individuals using kinship coefficients estimated by KING^34^.

To quantify genetic affinities between the modern and ancient populations, we used the programs qp3Pop and qpDstat in the ADMIXTOOLS suite (https://github.com/DReichLab^35^ for f3- and f4-statistics, respectively. f3-statistics of the form f3(X,Y,Outgroup) measure the amount of shared genetic drift of populations X and Y after their divergence from an outgroup. We used an ancient population and a modern Caucasian population for X, Y and Mbuti as an outgroup. f4-statistics of the form f4(Outgroup,Test;X,Y) show if population Test is equally related to X and Y or shares an excess of alleles with either of the two. In the f4-statistic calculation we used Mbuti for Outgroup, a modern population of the Caucasus for Test, and X and Y for contemporaneous ancient populations. This meant that f4 < 0 indicated higher genetic affinity between the test population and X, while f4 > 0 indicated higher genetic affinity between the test population and Y.

To quantify geographic features, we derived least-cost paths and measured least-cost distances (LCD) between the modern and ancient populations using the Least Cost Path Plugin for QGIS. The computation of LCD considers a friction grid that is a raster map where each cell indicates the relative difficulty (or cost) of moving through that cell. A least-cost path minimizes the sum of frictions of all cells along the path, and this sum is the least-cost distance (LCD). For impedance to human movement and expansion, we used 15 geographic features (Table 3). All gridded geographic features (i.e. raster layers) were resampled to a resolution of 1 km using the nearest-neighbor assignment technique. All possible subsets of the 15 geographic features, that did not cancel out each other, were used to calculate different variables of LCD. We assumed that most human movements occurred during climate warming events when the earth’s surface was not dramatically different from that of today, and hence used the current data of the geographic features.

**Table 3:** Geographic features used in combinations to calculate least-cost distances (LCD) between ancient populations and modern Caucasians.

N	Geographic feature	Explanation	Cost value
1	Land	Land surface extracted from MODIS/Terra Land Water Mask Derived from MODIS and SRTM L3 Yearly Global 250 m SIN Grid (MOD44W)^a^	1
2	Terrain Ruggedness Index (TRI)	TRI, calculated from the SRTM 1-km elevation grid as the mean difference between a central pixel and its surrounding cells using QGIS Desktop 3.10.6-A Coruña	TRI + 1
3	River	Class: 0, IGBP (Type 1): Water surface, extracted from MODIS/Terra + Aqua Land Cover Type Yearly L3 Global 500 m SIN Grid (MCD12Q1)^a^	Full barrier
4	River	Class: 0, IGBP (Type 1): Water surface, extracted from MODIS/Terra + Aqua Land Cover Type Yearly L3 Global 500 m SIN Grid (MCD12Q1)^a^	TRI + 1
5	Glacier	Class: 15, IGBP (Type 1): Permanent snow and ice, extracted from MODIS/Terra + Aqua Land Cover Type Yearly L3 Global 500 m SIN Grid (MCD12Q1)^a^	Full barrier
6	Glacier	Class: 15, IGBP (Type 1): Permanent snow and ice, extracted from MODIS/Terra + Aqua Land Cover Type Yearly L3 Global 500 m SIN Grid (MCD12Q1)^a^	max(TRI + 1) + 1
7	Desert	Class: 16, IGBP (Type 1): Barren or sparsely vegetated, extracted from MODIS/Terra + Aqua Land Cover Type Yearly L3 Global 500 m SIN Grid (MCD12Q1)^a^	Full barrier
8	Desert	Class: 16, IGBP (Type 1): Barren or sparsely vegetated, extracted from MODIS/Terra + Aqua Land Cover Type Yearly L3 Global 500 m SIN Grid (MCD12Q1)^a^	max(TRI + 1) + 1
9	Swamp	Class: 11, IGBP (Type 1): Permanent wetlands, extracted from MODIS/Terra + Aqua Land Cover Type Yearly L3 Global 500 m SIN Grid (MCD12Q1)^a^	Full barrier
10	Swamp	Class: 11, IGBP (Type 1): Permanent wetlands, extracted from MODIS/Terra + Aqua Land Cover Type Yearly L3 Global 500 m SIN Grid (MCD12Q1)^a^	max(TRI + 1) + 1
11	Bosporus-Dardanelles	A chain of natural straits connecting the Black Sea with the Aegean and Mediterranean seas	Full barrier
12	Bosporus-Dardanelles	A chain of natural straits connecting the Black Sea with the Aegean and Mediterranean seas	1
13	English Channel	A natural strait separating Southern England from northern France	Full barrier
14	English Channel	A natural strait separating Southern England from northern France	1
15	Riversides in desert	Lline and polygon networks of rivers and lakes were taken from (1) HydroSHEDS developed by the Conservation Science Program of the WWF (https://www.hydrosheds.org) and (2) the VMAP0 dataset from the National Imagery and Mapping Agency’s (NIMA) Digital Chart of the World (DCW) (http://www.mapability.com)	TRI + 1

^a^Obtained from https://search.earthdata.nasa.gov/search.

### Linking genetic affinity and geography

Generalized additive models (GAMs) were used to fit the outgroup f3-statistic to time and variously calculated LCD between the modern and ancient populations using the “mgcv” package^36^ in R version 3.5.2. Time between the modern and ancient populations was measured in BP (years before present, defined by convention as years before 1950 CE). We used GAMs because without any assumptions they are able to find nonlinear and non-monotonic relationships. GAMs were fitted using a Gamma family with a log link function. Penalized thin plate regression splines were used to represent all the smooth terms. The restricted maximum likelihood (REML) estimation method was implemented to estimate the smoothing parameter because it is the most robust of the available GAM methods^36^.

Model and variable selection were performed by exploring LCD, time BP and the interaction term. The predictive power of the models was evaluated through a tenfold cross-validation. The cross-validation of many models was handled through R’s parallelization capabilities^37,38^. The best model was selected by the mean squared error of the cross-validation. Akaike’s Information Criterion (AIC) is generally used as a means for model selection. However, we preferred cross-validation for model selection because AIC a priori assumes that simpler models with the high goodness of fit are more likely to have the higher predictive power, while cross-validation without any a priori assumptions measures the predictive performance of a model by efficiently running model training and testing on the available data.

We additionally validated the effect of different subsets of geographic features by assessing the relationship between statistically significant values of f4-statistic (i.e. |Z|> 3) and each subset. The relationship between f4-statistic of the form of f4(Outgroup,Test;X,Y) and geographic features was determined by measuring the agreement between the negative/positive signs of f4-statistic and the difference in LCD (LCD.D) for each pair of contemporaneous ancient populations X and Y. LCD.D was calculated as (LCD1–LCD2), where LCD1 was least-cost distance between the test population and X, and LCD2 was least-cost distance between the test population and Y. LCD.D < 0 indicated less least-cost distance between Test and X, while LCD.D > 0 indicated less least-cost distance between Test and Y. So, the same sign of f4 and LCD.D values indicated agreement between geographic proximity and genetic affinity. We used Cohen’s kappa^39^ to measure the agreement.

In order to test if geographic features (Table 3) accounted for present-day genetic differentiation in the Caucasus, we measured the relationship between Fst and LCD across the modern populations using the Mantel test in the “vegan” package^40^ in R version 3.5.2. In addition, we checked whether contribution from ancient samples was related to today’s genetic differentiation. To do so, we calculated median of f3-statistic of ancient populations of each geographic grouping (e.g. the following 6 populations made up one group: Balkans 39,950–41,950 BP, Balkans 37,950–39,950 BP, Balkans 31,950–33,950 BP, Balkans 9950–11,950 BP, Balkans 7950–9950 BP, Balkans 5950–7950 BP). Then we measured the manhattan distance of f3 median values of all combinations of the geographic groupings between the modern populations and compared the results to Fst and LCD using the Mantel test.

## Results

12 sampled individuals were inferred to be 3rd and 4th degree relatives, while the rest were unrelated: two 3rd degree relatives from CHCHN, four 4th degree relatives from KHEVS, two 4th degree relatives from LAZ, two 4th degree relatives from MSKH and two 4th degree relatives from TUSH. Our sample of modern populations showed near-zero levels of inbreeding (Table 4). Average Fst between the modern populations in the Caucasus was 0.00951, with the maximum value of 0.027 between KHEVS and MSKH (Table 5). There was no significant differentiation between Kartvelian speakers AJAR, SMG, IMR, KRT and KAKH (Z < 3). The rest were significantly different from these and each other. ADMIXTURE analyses on the modern data distinguished KHEVS and TUSH from the rest across all K values (Supplementary Fig. S1). K = 2 had the smallest of the cross-validation errors that increased with K. The ADMIXTURE plots suggested a cline running from southwest and south (LAZ, MSKH) to north and northeast (CHCHN, KHEVS, TUSH).

**Table 4:** Statistics of excess homozygosity-based inbreeding estimate (“fhat2” estimate of PLINK 1.9).

Population	Sample size	Average	Median	SD
AJAR	3	− 0.0099	− 0.01159	0.006362
ARM	6	− 0.00768	− 0.01027	0.007899
BLK	4	− 0.01901	− 0.01717	0.008596
CHCHN	6	− 0.01316	− 0.01504	0.005919
IMR	5	− 0.00464	− 0.0046	0.007384
KAKH	5	− 0.01996	− 0.01789	0.008032
KHEVS	4	0.025342	0.01801	0.024001
KRCH	3	− 0.0245	− 0.02507	0.012574
KRT	6	− 0.01413	− 0.01487	0.004248
LAZ	11	0.003605	0.003279	0.011749
MSKH	5	− 0.00034	− 6.72E−05	0.011031
SMG	5	− 0.00399	− 0.00777	0.009283
SVN	5	0.006769	0.003519	0.009194
TUSH	5	0.008185	0.007489	0.012841

**Table 5:** Matrix of Fst (upper triangular) and Z-values (lower triangular). Number of samples used: 73; number of SNPs used: 138,782; number of blocks for moving block jackknife: 692.

	AJAR	ARM	BLK	CHCHN	IMR	KAKH	KHEVS	KRCH	KRT	LAZ	MSKH	SMG	SVN	TUSH
AJAR	0	0.003	0.005	0.008	0.001	0.002	0.021	0.007	0	0.003	0.008	0	0.003	0.013
ARM	4.356	0	0.007	0.01	0.005	0.002	0.024	0.01	0.003	0.006	0.007	0.005	0.01	0.016
BLK	5.486	9.999	0	0.007	0.005	0.004	0.022	0.003	0.004	0.008	0.011	0.004	0.007	0.015
CHCHN	9.999	9.999	9.999	0	0.009	0.007	0.024	0.01	0.007	0.012	0.014	0.009	0.012	0.016
IMR	1.029	9.392	6.495	9.999	0	0.001	0.021	0.007	0.001	0.004	0.009	0	0.004	0.014
KAKH	2.132	4.09	5.567	9.999	1.342	0	0.018	0.006	− 0.001	0.004	0.006	0.001	0.005	0.011
KHEVS	9.999	9.999	9.999	9.999	9.999	9.999	0	0.026	0.016	0.025	0.027	0.022	0.025	0.013
KRCH	6.567	9.999	3.412	9.999	8.028	7.128	9.999	0	0.007	0.012	0.015	0.007	0.009	0.017
KRT	0.241	5.725	6.815	9.999	1.024	− 2.523	9.999	7.993	0	0.003	0.007	0.001	0.005	0.01
LAZ	4.205	9.999	9.999	9.999	9.077	8.666	9.999	9.999	9.248	0	0.011	0.004	0.008	0.017
MSKH	9.124	9.999	9.999	9.999	9.999	9.811	9.999	9.999	9.999	9.999	0	0.009	0.014	0.02
SMG	0.506	9.887	5.304	9.999	0.362	2.053	9.999	7.464	2.188	8.597	9.999	0	0.003	0.015
SVN	3.725	9.999	9.835	9.999	6.879	8.708	9.999	9.611	8.318	9.999	9.999	4.178	0	0.017
TUSH	9.999	9.999	9.999	9.999	9.999	9.999	9.999	9.999	9.999	9.999	9.999	9.999	9.999	0

The first and second PCA axes (Supplementary Fig. S2) showed a clear gradient from northeastern Anatolia (Kartvelian-speaking Laz communities) to the northern (Balkar, Karachay and Chechen communities) and northeastern (Kartvelian-speaking Tush and Khevsur) Caucasus. Populations occurring south of the main ridge of the Greater Caucasus were more closely related to each other than the northern populations were to each other. The northern populations showed two distinct clusters: the northeastern cluster of Kartvelian speakers (KHEVS, TUSH) and the northern cluster of Northeast-Caucasian and Turkic speakers (CHCHN, BLK, KRCH). Projecting the ancient populations onto the first two PCA axes generated a similar gradient, where the ancient populations of Anatolia and Levant were positioned opposite of the ancient Siberia and Europe. These gradients had the ancient populations of Levant-Anatolia and the modern populations of NE Anatolia at one end, and the ancient populations of Siberia and Europe and the modern populations of northern Caucasus at the other end. The ancient Caucasian hunter-gatherers fell within the variation of present-day Kartvelian speakers SVN and SMG. Ancient Anatolians and Levantines fell within the variation of southern Kartvelian speakers (MSKH) and Armenian speakers (ARM). Ancient Siberians and Europeans were closer to populations north of the Greater Caucasus main ridge (Chechens, Balkars and Karachays).

**Figure 2:**
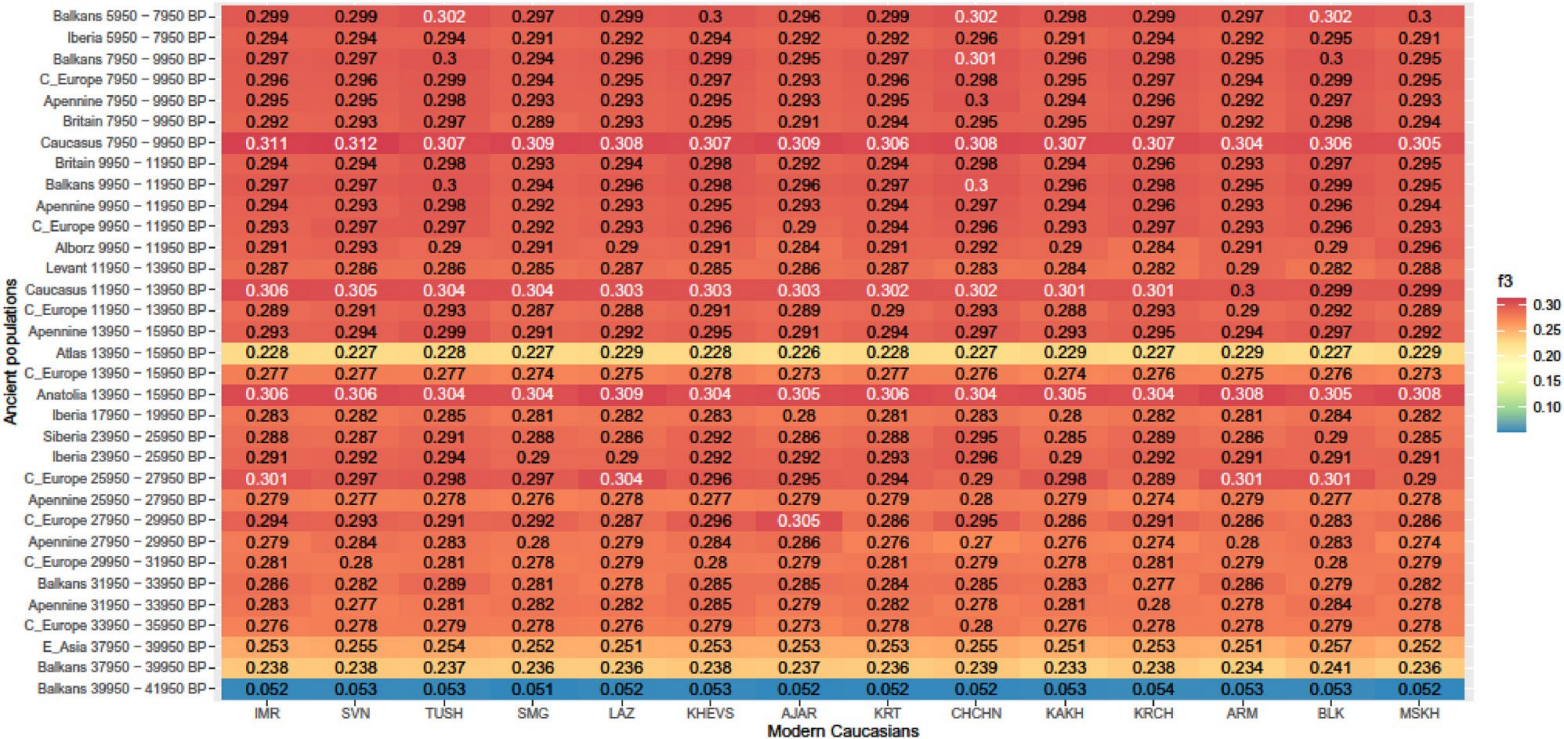
Genetic affinity (f3) between modern Caucasians and ancient populations.

**Figure 3:**
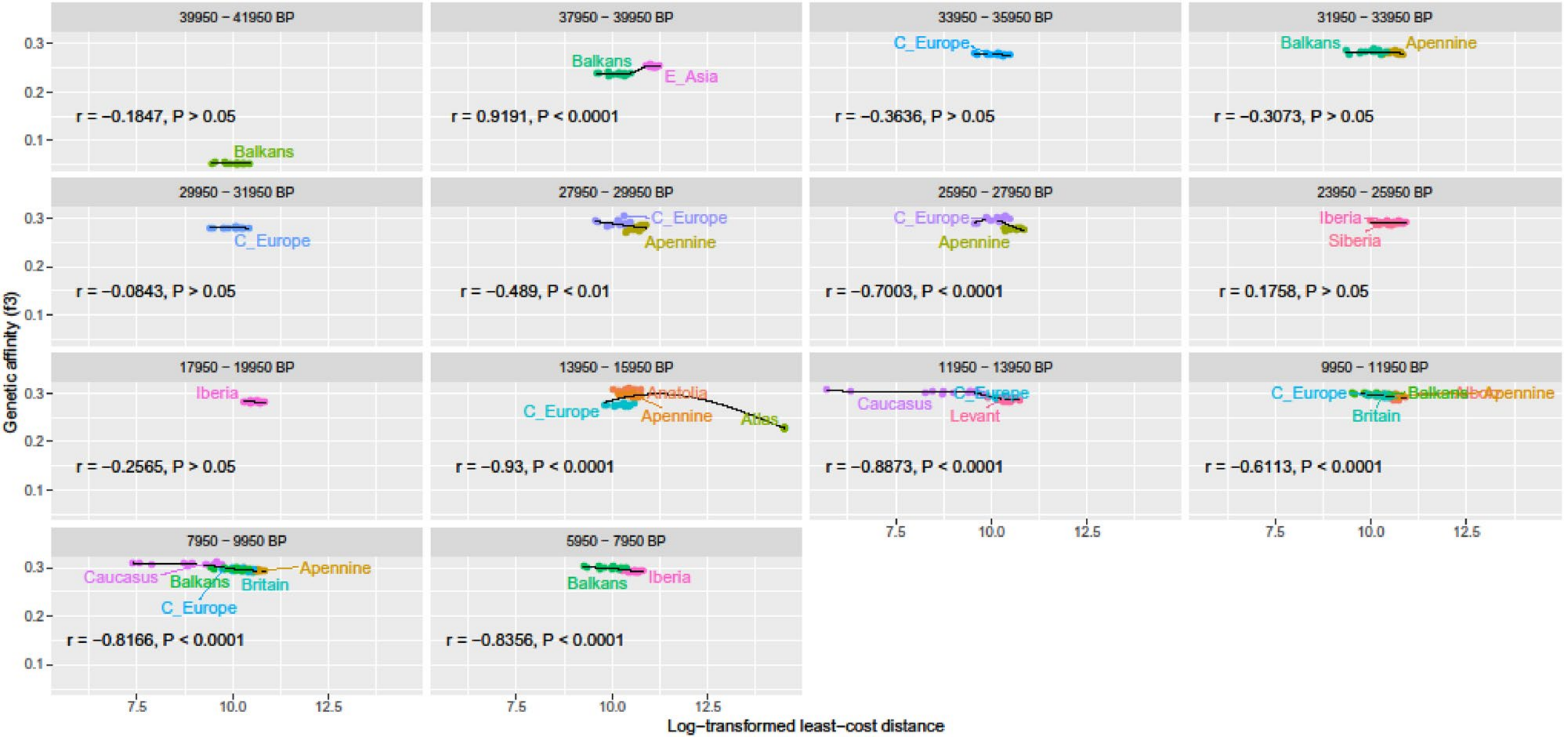
Genetic affinity (f3) between modern populations of the Caucasus and ancient populations (labeled in different colors), plotted against least-cost distance for different 2000-year-long periods before present (BP). Pearson correlation coefficients (r) show relationships between f3 and linear least-cost distance. The smoothing lines are applied using the GAM-REML method.

Generally, modern Caucasian populations had more genetic affinity with those ancient populations that were closer in time and space, and this relationship well explained the genetic difference between the modern populations (Figs. 2, 3, Supplementary Figs. S2–S5). This affinity was highest with the early post-glacial populations of the Caucasus, Anatolia and the Balkans. The ancient Caucasian ancestry was the highest in Kartvelian-speaking groups peaking in Imeretians (IMR), Svans (SVN) and Megrelians (SMG) in western Georgia—that is, the populations in the closest geographic proximity to the findings of the ancient Caucasian hunter-gatherers (aka CHG). Anatolian ancestry was the highest in southern and south-western Kartvelian-speaking Meskhs and Lazs (MSKH, LAZ) and Indo-European-speaking Armenians (ARM) in southern Georgia and north-eastern Turkey, respectively. Balkan and Siberian ancestry peaked in groups occurring north of the main ridge of the Greater Caucasus, namely in Kartvelian-speaking Tushs (TUSH) in north-eastern Georgia, Northeast-Caucasian-speaking Chechens (CHCHN) and Turkic-speaking Balkars (BLK) in the Russian Federation.

**Figure 4:**
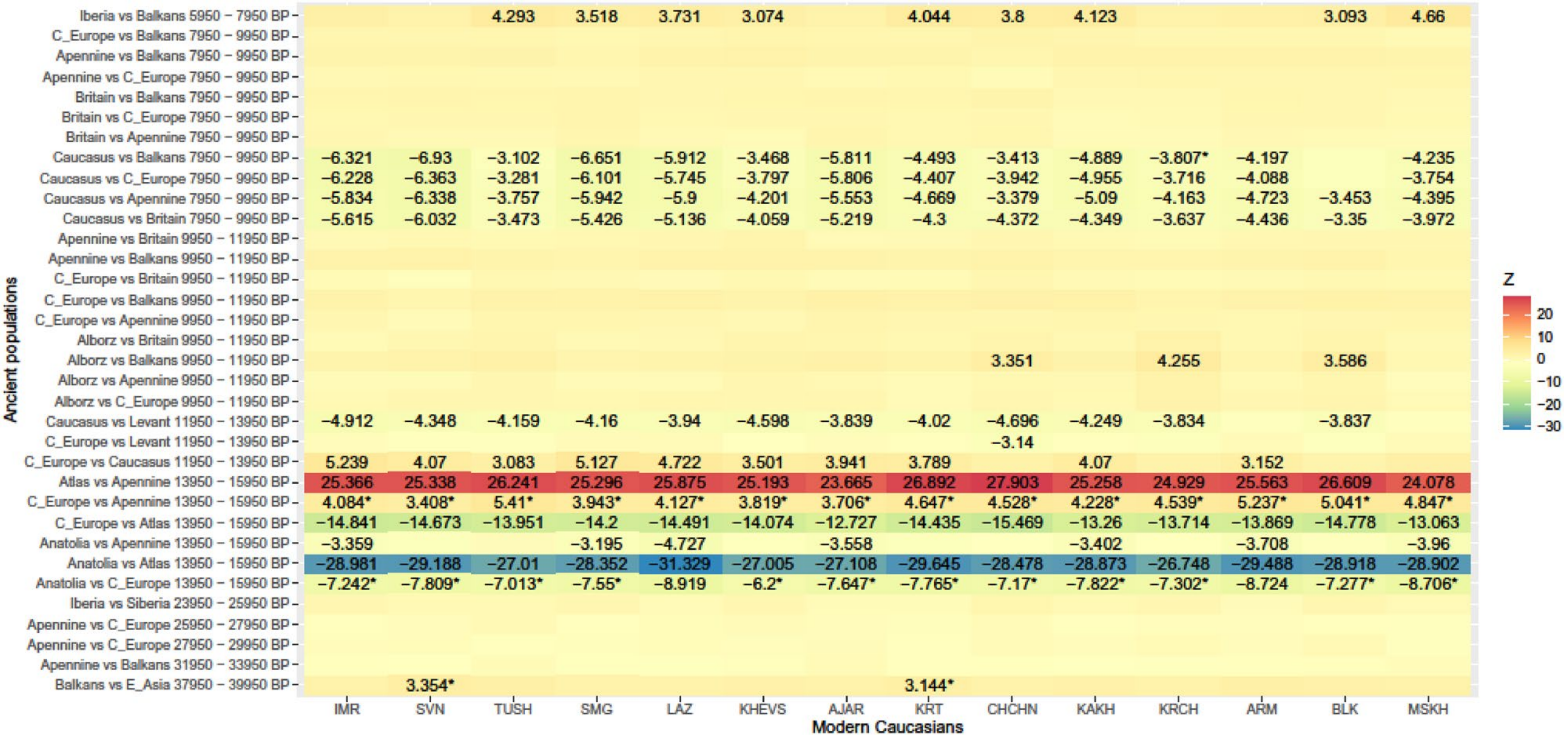
Genetic affinity (f4) between modern populations of the Caucasus and pairs of contemporaneous ancient populations. The numbers show the significant Z-values of f4 (i.e. |Z|> 3). Z < 0 indicates higher genetic affinity between the modern population and the ancient population at left of the pair on the Y-axis, while Z > 0 indicates higher genetic affinity between the modern population and the ancient population at right. Asterisk indicates disagreement between the genetic affinity and geographic proximity based on the least-cost distance.

Genetic affinity of f3-statistic between the modern Caucasians and ancient populations was best explained by the interaction of LCD and mean time BP between these populations (Table 6). In the best model LCD implied that (1) human movement was impeded by terrain ruggedness (TRI), (2) the Bosporus-Dardanelles and the English Channel did not act as barriers, (3) swamps, glaciers and desert were not full barriers, but permeable at the highest cost of the cost grid, and (4) riversides in desert and rivers were permeable at TRI values—i.e., the cost grid was the combination of geographic features 2, 4, 6, 8, 10, 12, 14 and 15 identified in Table 3. Genetic affinity generally increased as time BP and LCD decreased (Fig. 3, Supplementary Figs. S3 and S4). This cost grid generated LCD.D that had the greatest measure of agreement with genetic affinity based on f4-statistic (Kappa = 0.59, p-value = 2.02e−14, Fig. 4). There was overall negative correlation between f3-statistic and LCD (r = − 0.207, p < 0.0001), and between f3-statistic and time BP (r = − 0.540, p < 0.0001). Semi-partial correlation between f3 and LCD (controlled for the influence of time BP on f3) was − 0.297 (p < 0.0001). Correlation between LCD and genetic affinity were highly significant for a period of 5950–15,950 BP (r = − 0.909, p < 0.0001). During the glacial peak (17,950–25,950 BP) the correlation decreased (r = − 0.032, p = 0.8412). Further back in time (i.e. time BP > 25,950) the correlation was mostly not significant.

**Table 6:** Summary of the generalized additive model (GAM) analysis for modeling the genetic affinity (f3) between the modern Caucasians and ancient populations in relation to standardized values of least-cost distance (LCD) and mean time BP. n = sample size; s() = spline smooth function with interaction between the two variables; e.d.f. = estimated degrees of freedom; *p* = significance of terms.

n	Variable term	e.d.f	*p*	Goodness of fit (R^2^_adj_)	Deviance explained
490	s(LCD, BP_mean)	28.89	< 2e−16	0.991	99.6%
	Intercept = − 1.3051265		< 2e−16

Our model had disagreement between genetic affinity and geographic proximity (as a function of landscape permeability) to ancient populations dating from 13,950 to 15,950 BP and 37,950–39,950 BP (Figs. 3 and 4). All modern samples of the Caucasus had more affinity with Apennine 13,950–15,950 BP than with C. Europe 13,950–15,950 BP that was at closer least-cost distance, and all modern samples northeast of Laz and Armenians had more affinity with Anatolia 13,950–15,950 BP than with C. Europe 13,950–15,950 BP that was at closer least-cost distance. There was more affinity of our sample of modern Caucasians with ancient E. Asian individual than with a Balkan individual 37,950–39,950 BP that was at closer least-cost distance.

**Figure 5:**
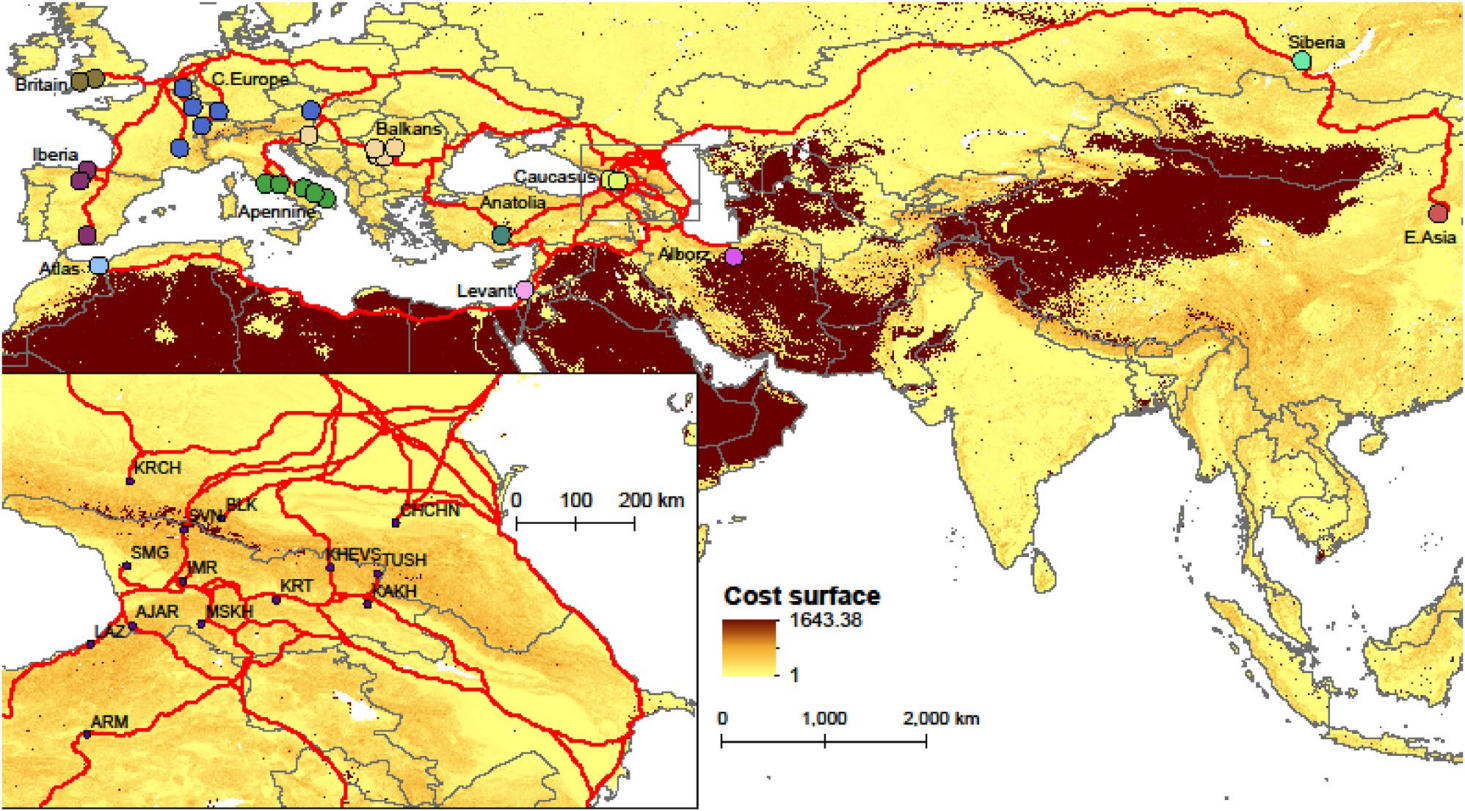
Migration pathways that best explain genetic affinity between modern Caucasians and ancient humans, showing least-cost paths from Eurasian and African ancient populations (shown in the main map, see Table 2 for details) to the current Caucasian populations (identified in the inset map, see Table 1 for details). The least-cost paths imply that (1) human movement is impeded by terrain ruggedness (TRI), (2) the Bosporus-Dardanelles and the English Channel do not act as barriers, (3) swamps, glaciers and desert are not full barriers, but permeable at the highest of the cost grid values, and (4) riversides in desert are permeable at TRI values. It is assumed that most human movements occurred during climate warming events when the earth’s surface was not dramatically different from that of today, and hence the current data of the geographic features are used in the calculation of the least-cost paths. The map is generated using QGIS Desktop 3.10.6-A Coruña (https://qgis.org).

Our model generated least-cost paths from Eurasian and African ancient populations to the current Caucasian populations (Fig. 5). All least-cost paths from ancient populations of Europe, Siberia and East Asia reached populations south of the Greater Caucasus through the East European Plain, the north-western Caspian coast and the flood plain of the River Mtkvari (aka Kura) (Supplementary Fig. S6). All ancient populations occurring south of the Greater Caucasus reached populations north of the Greater Caucasus through either the western Caspian coast or across the Bosporus-Dardanelles and through the East European Plain (Supplementary Fig. S6). Least-cost paths from the ancient population of Anatolia to the Caspian watershed of the Caucasus ran across the Bosporus-Dardanelles and through the East European Plain, the western Caspian coast and the flood plain of the River Mtkvari (Supplementary Fig. S6). Least-cost paths from Africa, Levant, Anatolia and Alborz reached the southern Caucasus either across the Pontic Mountains or through the flood plains of the Rivers Araxes and Mtkvari. Least-cost paths from Africa converged in the Levant before reaching the Caucasus.

Fst had the strongest correlation with LCD derived from the same geographic features that explained best f3 and f4 statistics (Mantel Test: r = 0.402, Monte-Carlo simulated p-value = 0.0295 based on 1,000,000 replicates; Supplementary Fig. S7). Neighbor-Joining tree based on Fst (Supplementary Fig. S8) clustered together (1) Kartvelian-speaking populations from western Georgia and north-eastern Turkey, (2) Kartvelian and Armenian speakers from southern Georgia and eastern Turkey, (3) Northeast-Caucasian and Turkic speakers from the north Caucasus, and (4) Kartvelian speakers from the northeastern Greater Caucasus being the most genetically distinct from the others. Sampled Kartvelian-speaking populations from central (KRT) and eastern Georgia (KAKH) were most admixed. Additional Mantel tests showed that Fst was significantly correlated with difference in contribution from a combination of 4 ancient populations: Anatolia, Balkans, Caucasus, Siberia (r = 0.2906, p = 0.04033). The difference in contribution from these ancient populations was also significantly correlated with LCD (r = 0.3701, p = 0.003187). Partial Mantel test controlling for LCD made the correlation between Fst and ancient contribution insignificant (r = 0.1668, p = 0.15608). Controlling for ancient contribution significantly reduced the correlation between Fst and LCD (r = 0.311, p = 0.1). The Mantel tests suggested that LCD and ancient ancestry explained much of the present-day genetic variation in the Caucasus.

## Discussion

Our results suggest that the current populations of the Caucasus bear detectable ancestry from Caucasian, Anatolian and Balkan hunter-gatherers. Caucasian hunter-gatherers (CHG) are the major gene contributors to the modern Caucasian populations. The proportion of CHG alleles is the highest in modern populations that live in close proximity to the archaeological sites in western Georgia, where the bones of CHG were discovered^8^, and gradually decrease away from this area, being replaced mostly with alleles of ancient Anatolians and Europeans. These archaeological sites fall within the Colchic refugium where humans survived a series of glacial maxima including the LGM in the Caucasus^5^. Our model shows that in early post-glacial time the migration of hunter-gatherers from elsewhere into the Caucasus intensified. Ancient Anatolian alleles are most frequent in the genomes of modern people from southern Georgia and eastern Turkey (i.e. Georgians from Meskheti province, Laz and Armenians), while ancient European or Balkan alleles are most common in modern populations of the North Caucasus, suggesting that European hunter-gatherers migrated to the Caucasus across the East European Plain rather than through Asia Minor. Remarkably, ancient genomes from Levant (Natufian culture) and Atlas Mountains (northwestern Africa), have the highest allele share with the same modern populations as ancient Anatolians, which suggests some admixture between these populations before Anatolian population started to expand to the Caucasus. The similarity of genetic gradients of the ancient and modern Caucasus in our PCA plot once again confirms genetic continuity spanning from the Late Upper Paleolithic until today^8,25^. Our study explicitly explains the reasons behind this phenomenon.

Traces of different ancient populations (migration sources) in modern communities of the Caucasus decrease with (i) geographic distance weighted by physical barriers to dispersal, and (ii) the age of the ancient population. This signal is sufficiently strong to differentiate between the modern populations by their affinity to individual ancient populations. Our analyses suggest that the expansion of the refugial populations of Upper Paleolithic and Mesolithic hunter-gatherers was greatly impeded by rugged terrain, swamps, glaciers and desert, and the genetic legacy of these hunter-gatherers in the modern Caucasus is well explained by landscape permeability between the refugial human populations. The relationship between the ancient genetic signature and landscape permeability is stronger if the refugial populations are closer in time to the present-day Caucasus, suggesting that the genetic signature associated with a particular ancestral area fades away as a result of gene drift and later admixture events.

Although our model performed highly significantly, it failed to explain disagreement between genetic affinity and geographic proximity (as a function of landscape permeability) to ancient populations dating from 13,950–15,950 BP and 37,950–39,950 BP (Figs. 3 and 4). All modern samples of the Caucasus had more affinity with Apennine 13,950–15,950 BP than with geographically closer C. Europe 13,950–15,950 BP, and all modern samples northeast of Laz and Armenians had more affinity with Anatolia 13,950–15,950 BP than with geographically closer C. Europe 13,950–15,950 BP. This is probably due to early post-glacial admixture of Anatolian hunter-gatherers with Caucasian and European hunter-gatherers, and that of East Asian hunter-gatherers with European hunter-gatherers at this period^41,42^. These admixture events, coupled with our landscape permeability model, suggest that ancient Anatolians carrying the genetic signature of Caucasian hunter-gatherers would contribute more to Apennine hunter-gatherers through Bosporus-Dardanelles and the Balkans than to C. European ones, while C. European ones would get more genes through Eurasian Steppes from East Asian hunter-gatherers. These events of admixture from east to west, that may be facilitated by technological advances or advantages through natural selection (e.g. resistance to lethal infectious diseases), make our sample of modern populations more closely related to the ancient populations from Anatolia and the Apennine than to the ancient population from C. Europe.

Our sample of ancient populations dating from 37,950 to 39,950 BP were each represented by a single individual from the Balkans and E. Asia. More affinity of our sample of modern Caucasians with an ancient E. Asian individual than with a geographically closer Balkan individual 37,950–39,950 BP could be explained by two scenarios: either this Balkan individual was a vagrant from elsewhere, which was shown not to have contributed to any present-day populations^43^ or between when anatomically modern humans arrived in Europe approximately 45,000 years ago^44^ and the LGM, climate was the mildest around 37,950–39,950 BP^45,46^ allowing more gene flow between western and eastern Eurasia. This gene flow is supported by the fact that this individual from the Balkans was more closely related to East Asians than Paleolithic and Mesolithic Europeans^47^, while a contemporaneous individual from western Russia was more closely related to later Europeans than to East Asians^48^. At glacial maxima human source populations were highly fragmented, had no or limited gene flow with each other and survived in glacial refugia, from where the populations would expand during benign climates and shrink back to during harsh climates^5^. Thus, our model better explains the genetic legacy of ancient populations in the modern Caucasus, if there is more genetic dissimilarity between these ancient populations than within them.

Even today genetic affinity (Fst) between modern populations of the Caucasus significantly correlates with least-cost distance between the hubs of these populations, which is in line with previous studies based on limited sample and genetic markers^22^. The genetic affinity between the modern populations has the strongest response to the least-cost distance weighted by the same geographic features that account best for the genetic affinity between the ancient and the modern populations. However, without considering the past metapopulation picture, landscape permeability between present-day populations fails to explain the considerable fraction of variation in current genetic structure in the Caucasus.

The derived least-cost paths suggested that both the Greater Caucasus, the Lesser Caucasus and the Pontic Mountains considerably impeded the inferred human movements through the Caucasus (Figs. 1, 5, Supplementary Fig. S6). The Greater Caucasus posed the major barrier to human movements that is in agreement with the north–south cline in genetic variation (this study^26,49^. The genetic differentiation was the strongest between the populations from south and north of the Greater Caucasus main ridge, which once again indicates that the Greater Caucasus has been an important physical barrier for human dispersal since the last glacial period. However, there were also noticeable differences between populations from the same side of this mountain range, suggesting their different exposure to incoming migrants through heterogenous landscape. The western flank of the Caspian Sea appeared to be more permeable for human movement than the eastern flank of the Black Sea. None of the least-cost paths from Europe, Siberia and East Asia ran through the eastern flank of the Black Sea. The eastern flank of the Black Sea is walled by the Greater Caucasus to the north and the Lesser Caucasus and the Pontic Mountains to the south, while the western flank of the Caspian Sea is largely open. This difference between the eastern and western edges of the Caucasus explains the east–west cline in Y-chromosome and autosomal genome variations in the Caucasus^7,17,22,24^.

We were not able to sample other ethnic groups inhabiting the Caucasus. These groups would have provided more information about the genetic structure of the region. However, our sample was sufficient to test our hypotheses. Publications to date have measured the gnome-wide ancestry of ancient populations in many of the present-day communities on earth. However, to the best of our knowledge, this study is the first of its kind to estimate the relationship of genome-wide genetic affinity between modern and ancient populations to landscape permeability as a function of different geographic features, and infer major dispersal paths from refugial populations. Our model can help to time and map the dispersal and migration paths of prehistoric events not only in relation to the Caucasus but also elsewhere. Presence of significant negative correlation between the weighted geographic distance from the ancient source of a distinct genetic signature and genetic affinity with this source can be seen as evidence of migration via a particular path and at a particular time.

## Supplementary Information

Supplementary Information.

## Data Availability

All data generated or analyzed during this study are included in this published article (and its Supplementary Information files). Any additional data related to this study are available from the corresponding author on request.
