# Supplementary material for: Landscape genetics and the genetic legacy of Upper Paleolithic and Mesolithic hunter-gatherers in the modern Caucasus
Source: Sci Rep. 2021 Sep 9;11:17985. doi: 10.1038/s41598-021-97519-6 (PMC8429691; doi:10.1038/s41598-021-97519-6)
Supplement: Supplementary file 1 — Supplementary Information. [file 41598_2021_97519_MOESM1_ESM.pdf]

Landscape genetics and the genetic legacy of Upper Paleolithic and Mesolithic hunter-gatherers in the modern Caucasus

Alexander Gavashelishvili *, Center of Biodiversity Studies, Institute of Ecology, Ilia State University, Cholokashvili Str. 5, 0162 Tbilisi (Georgia)
E-mail: aleksandre.gavashelishvili@iliauni.edu.ge

Alexey Yanchukov, Department of Biology, Faculty of Arts and Sciences, Bülent Ecevit University, Zonguldak, Turkey
E-mail: yawa33@gmail.com

David Tarkhnishvili, School of Natural Sciences and Engineering, Ilia State University, Tbilisi, Georgia
E-mail: david_tarkhnishvili@iliauni.edu.ge

Marine Murtskhvaladze, School of Natural Sciences and Engineering, Ilia State University, Tbilisi, Georgia, L. Sakvarelidze National Center for Disease Control and Public Health, Tbilisi, Georgia
E-mail: dna_lab@iliauni.edu.ge

Irakli Akhvlediani, Georgian FTDNA project, Calle Francisco Tierra N2, 9A., Barakalado 48903,Vizcaya,Spain.
E-mail: irakliakhvlediani1982@yahoo.com

Ceren Kazancı, Life Sciences, Institute of Ecology, Ilia State University, Cholokashvili Str. 5, 0162 Tbilisi (Georgia).
E-mail: ceren.kazanci.1@iliauni.edu.ge

* Corresponding author: Alexander Gavashelishvili

Table S1 – Modern Mbuti individuals genotyped for whole genome. These data are retrieved from David Reich's Lab website, Harvard University (https://reich.hms.harvard.edu/downloadable-genotypes-present-day-and-ancient-dna-data-compiled-published-papers).

Mbuti	HGDP00449	SGDP	male
Mbuti	HGDP00462	HGDP	male
Mbuti	HGDP00463	HGDP	male
Mbuti	HGDP00467	HGDP	male
Mbuti	HGDP00474	SGDP	male
Mbuti	HGDP00476	SGDP	female
Mbuti	HGDP00478	HGDP	male
Mbuti	HGDP00982	SGDP	male
Mbuti	HGDP00984	HGDP	male
Mbuti	HGDP01081	HGDP	male

Table S2 – Upper Paleolithic-Mesolithic anatomically modern human individuals genotyped for whole genome. These data are retrieved as a part of 1240K dataset from David Reich's Lab website, Harvard University (https://reich.hms.harvard.edu/downloadable-genotypes-present-day-and-ancient-dna-data-compiled-published-papers). The ancient genotypes are selected such that they either date from the LGM or fall within the glacial refugia identified by Gavashelishvili and Tarkhnishvili (2016) in order to maximize the genetic signature of potential refugial populations in our analysis. The ancient genotypes are divided into 2000-year-long intervals (column: BP_interval), and then each of these intervals is grouped into geographic units (column: Ancient populations).

Index	Master ID	Publication	Average age in calBP	Locality	Country	Latitude	Longitude	Sex	Ancient Population	BP_Interval
475	Vindija	Pruefer 2017	41950	Vindija Cave	Croatia	46.299167	16.070556	F	Balkans1	39950 - 41950
663	Oase1	Fu Nature 2015	39610	Oase Cave	Romania	45.12	21.9	M	Balkans2	37950 - 39950
1465	Tianyuan	Yang Current Biology 2017	39475	Tianyuan	China	39.39	115.52	M	E_Asia2	37950 - 39950
546	GoyetQ116-1	Fu Nature 2016	34795	Troisieme caverne of Goyet cave	Belgium	50.446	5.008	M	C_Europe4	33950 - 35950
1037	Muierii2	Fu Nature 2016	33300	Muierii 2	Romania	45.11	23.46	F	Balkans5	31950 - 33950
550	Paglicci133	Fu Nature 2016	32895	Apulia, Paglicci	Italy	41.65	15.61	M	Apennine5	31950 - 33950
4638	Cioclovina1	Fu Nature 2016	32435	Cioclovina	Romania	45.35	23.84	M	Balkans5	31950 - 33950
1047	I1577	Fu Nature 2016	30950	Krems-Wachtberg	Austria	48.41	15.59	M	C_Europe6	29950 - 31950
1038	Vestonice13	Fu Nature 2016	30870	Dolni Vestonice	Czechia	48.53	16.39	M	C_Europe6	29950 - 31950
1039	Vestonice15	Fu Nature 2016	30870	Dolni Vestonice	Czechia	48.53	16.39	M	C_Europe6	29950 - 31950
1044	Vestonice14	Fu Nature 2016	30870	Dolni Vestonice	Czechia	48.53	16.39	M	C_Europe6	29950 - 31950
1036	Pavlov1	Fu Nature 2016	30260	Dolni Vestonice	Czechia	48.53	16.39	M	C_Europe6	29950 - 31950
1034	Vestonice16	Fu Nature 2016	30010	Dolni Vestonice	Czechia	48.53	16.39	M	C_Europe6	29950 - 31950
1040	Vestonice43	Fu Nature 2016	30010	Dolni Vestonice	Czechia	48.53	16.39	M	C_Europe6	29950 - 31950
1041	Ostuni2	Fu Nature 2016	28975	Apulia, Ostuni	Italy	40.73	17.57	F	Apennine7	27950 - 29950
548	GoyetQ53-1	Fu Nature 2016	27975	Troisieme caverne of Goyet cave	Belgium	50.446	5.008	F	C_Europe7	27950 - 29950
547	Paglicci108	Fu Nature 2016	27750	Apulia, Paglicci	Italy	41.65	15.61	F	Apennine8	25950 - 27950
1042	Ostuni1	Fu Nature 2016	27620	Apulia, Ostuni	Italy	40.73	17.57	F	Apennine8	25950 - 27950
4641	GoyetQ376-19	Fu Nature 2016	27515	Troisieme caverne of Goyet cave	Belgium	50.446	5.008	M	C_Europe8	25950 - 27950
549	GoyetQ56-16	Fu Nature 2016	26320	Troisieme caverne of Goyet cave	Belgium	50.446	5.008	F	C_Europe8	25950 - 27950

5438	I10899	Olalde Science 2019	24450	Granada, Carihuela Cave	Spain	37.4488	-3.4297	M	Iberia9	23950 - 25950
868	MA1	Raghavan Nature 2013	24305	Mal'ta	Russia	52.9	103.5	M	Siberia9	23950 - 25950
1045	ElMiron	Fu Nature 2016	18720	El Miron	Spain	43.26	-3.45	F	Iberia12	17950 - 19950
551	Rigney1	Fu Nature 2016	15465	Rigney	France	47.23	6.1	F	C_Europe14	13950 - 15950
5921	ZBC	Feldman Nature Communications 2019	15405	P?narbas?I	Turkey	37.483333	33.033333	M	Anatolia14	13950 - 15950
4642	HohleFels49	Fu Nature 2016	15130	Swabian Jura, Baden-Wurttemberg, Hohle Fels, Ach Valley	Germany	48.22	9.45	M	C_Europe14	13950 - 15950
5918	GoyetQ2	Villalba Mouco Current Biology 2019	15006	Troisieme caverne of Goyet cave	Belgium	50.446	5.008	M	C_Europe14	13950 - 15950
4639	Brillenhohle	Fu Nature 2016	14780	Swabian Jura, Baden-Wurttemberg, Brillenhöhle, Ach Valley	Germany	48.24	9.46	M	C_Europe14	13950 - 15950
4637	HohleFels79	Fu Nature 2016	14670	Swabian Jura, Baden-Wurttemberg, Hohle Fels, Ach Valley	Germany	48.22	9.45	M	C_Europe14	13950 - 15950
552	Burkhardtshohle	Fu Nature 2016	14615	Swabian Jura, Baden-Wurttemberg, Burkhardtshöhle, Westerheim	Germany	48.32	9.35	M	C_Europe14	13950 - 15950
4919	TAF010	vande Loosdrecht Science 2018	14605	Taforalt	Morocco	34.807812	-2.410889	M	Atlas14	13950 - 15950
4920	TAF011	vande Loosdrecht Science 2018	14500	Taforalt	Morocco	34.807812	-2.410889	M	Atlas14	13950 - 15950

4922	TAF013	vande Loosdrecht Science 2018	14500	Taforalt	Morocco	34.807812	-2.410889	M	Atlas14	13950 - 15950
4923	TAF014	vande Loosdrecht Science 2018	14500	Taforalt	Morocco	34.807812	-2.410889	M	Atlas14	13950 - 15950
4924	TAF015	vande Loosdrecht Science 2018	14500	Taforalt	Morocco	34.807812	-2.410889	M	Atlas14	13950 - 15950
4921	TAF012	vande Loosdrecht Science 2018	14470	Taforalt	Morocco	34.807812	-2.410889	F	Atlas14	13950 - 15950
4918	TAF009	vande Loosdrecht Science 2018	14357	Taforalt	Morocco	34.807812	-2.410889	M	Atlas14	13950 - 15950
1077	I2158	Mathieson Nature 2018	14275	Sicily, Egadi Islands, Favignana Island, Grotta d?¢,Ç¨,Ñ¢Oriente	Italy	37.933333	12.333333	F	Apennine14	13950 - 15950
1032	Villabruna	Fu Nature 2016	13980	Veneto, Villabruna	Italy	46.15	12.21	M	Apennine14	13950 - 15950
1109	Bichon	Jones Nature Communications 2015	13665	Grotte du Bichon	Switzerland	47.099998	6.8699999	M	C_Europe15	11950 - 13950
4578	I1687	Lazaridis Nature 2016	13265	Raqefet Cave	Israel	32.65	35.067	F	Levant15	11950 - 13950
1111	SATP	Jones Nature Communications 2015	13255	Satsurblia	Georgia	42.38	42.59	M	Caucasus15	11950 - 13950
553	Rochedane	Fu Nature 2016	12960	Rochedane	France	47.21	6.45	M	C_Europe15	11950 - 13950
1015	I0861	Lazaridis Nature 2016	12750	Raqefet Cave	Israel	32.65	35.067	M	Levant15	11950 - 13950
1108	I1072	Lazaridis Nature 2016	12750	Raqefet Cave	Israel	32.65	35.067	M	Levant15	11950 - 13950
4572	I1069	Lazaridis Nature 2016	12750	Raqefet Cave	Israel	32.65	35.067	M	Levant15	11950 - 13950
4575	I1685	Lazaridis Nature 2016	12750	Raqefet Cave	Israel	32.65	35.067	M	Levant15	11950 - 13950
4579	I1690	Lazaridis Nature 2016	12750	Raqefet Cave	Israel	32.65	35.067	M	Levant15	11950 - 13950

6653	I2312	Narasimhan Patterson Science 2019	11950	Alborz Mountains, near Behshahr, Belt Cave	Iran	35.59	53.5	M	Alborz16	9950 - 11950
6430	R11	Antonio Gao Moots Science 2019	11908	Grotta Continenza	Italy	41.96	13.54	M	Apennine16	9950 - 11950
572	Iboussieres25-1	Mathieson Nature 2018	11725	Aven des Iboussières à Malataverne, Rhône-Alpes	France	44.29	4.46	M	C_Europe16	9950 - 11950
573	Iboussieres31-2	Mathieson Nature 2018	11725	Aven des Iboussières à Malataverne, Rhône-Alpes	France	44.29	4.46	M	C_Europe16	9950 - 11950
554	Iboussieres39	Fu Nature 2016	11725	Aven des Iboussières à Malataverne, Rhône-Alpes	France	44.29	4.46	M	C_Europe16	9950 - 11950
1525	I4657	Mathieson Nature 2018	11465	Vlasac	Serbia	44.53368	22.05032	F	Balkans16	9950 - 11950
4184	I5241	Mathieson Nature 2018	11196	Padina	Serbia	44.595879	22.010568	F	Balkans16	9950 - 11950
1043	Continenza	Fu Nature 2016	10855	Abruzzo, Grotta de Continenza	Italy	41.96	13.54	F	Apennine16	9950 - 11950
4178	I5235	Mathieson Nature 2018	10835	Padina	Serbia	44.595879	22.010568	M	Balkans16	9950 - 11950
4183	I5240	Mathieson Nature 2018	10805	Padina	Serbia	44.595879	22.010568	M	Balkans16	9950 - 11950
1053	I1293	Lazaridis Nature 2016	10800	Alborz Mountains, near Behshahr, Hotu Cave	Iran	35.591	53.501	M	Alborz16	9950 - 11950
4186	I5244	Mathieson Nature 2018	10785	Padina	Serbia	44.595879	22.010568	F	Balkans16	9950 - 11950
6533	R7	Antonio Gao Moots Science 2019	10682	Grotta Continenza	Italy	41.96	13.54	M	Apennine16	9950 - 11950

4484	I6745	Brace Diekmann Nature Ecology Evolution 2019	10555	England, Somerset, Burrington Combe, Aveline's Hole	Great Britain	51.324706	-2.753248	F	Britain16	9950 - 11950
4185	I5242	Mathieson Nature 2018	10530	Padina	Serbia	44.595879	22.010568	F	Balkans16	9950 - 11950
4483	I6744	Brace Diekmann Nature Ecology Evolution 2019	10399	England, Somerset, Burrington Combe, Aveline's Hole	Great Britain	51.324706	-2.753248	M	Britain16	9950 - 11950
4182	I5239	Mathieson Nature 2018	10333	Padina	Serbia	44.595879	22.010568	F	Balkans16	9950 - 11950
6575	I6767	Brace Diekmann Nature Ecology Evolution 2019	10300	England, Somerset, Cheddar, Gough's Cave	Great Britain	51.281679	-2.765746	M	Britain16	9950 - 11950
4645	Ranchot88	Fu Nature 2016	10085	Ranchot	France	47.91	5.43	F	C_Europe16	9950 - 11950
4244	I5773	Mathieson Nature 2018	10040	Vlasac	Serbia	44.53368	22.05032	M	Balkans16	9950 - 11950
4179	I5236	Mathieson Nature 2018	10008	Padina	Serbia	44.595879	22.010568	M	Balkans16	9950 - 11950
4181	I5238	Mathieson Nature 2018	9993	Padina	Serbia	44.595879	22.010568	F	Balkans16	9950 - 11950
4636	LesCloseaux13	Fu Nature 2016	9900	Les Closeaux	France	48.52	2.11	M	C_Europe17	7950 - 9950
4389	I5407	Mathieson Nature 2018	9800	Lepenski Vir	Serbia	44.552924	22.027563	F	Balkans17	7950 - 9950
4177	I5234	Mathieson Nature 2018	9800	Padina	Serbia	44.595879	22.010568	F	Balkans17	7950 - 9950
4180	I5237	Mathieson Nature 2018	9800	Padina	Serbia	44.595879	22.010568	M	Balkans17	7950 - 9950
4391	I5409	Mathieson Nature 2018	9800	Padina	Serbia	44.595879	22.010568	F	Balkans17	7950 - 9950
1110	KK1	Jones Nature Communications 2015	9720	Kotias Klde	Georgia	42.28	43.28	M	Caucasus17	7950 - 9950
4598	I4660	Mathieson Nature 2018	9713	Vlasac	Serbia	44.53368	22.05032	F	Balkans17	7950 - 9950
4512	I6754	Brace Diekmann Nature Ecology Evolution 2019	9588	Wales, Pembrokeshire, Caldey Island, Ogof-Yr-Ychen	Great Britain	51.63253	-4.692903	M	Britain17	7950 - 9950

1483	I4081	Mathieson Nature 2018	9335	Ostrovul Corbului	Romania	44.517	22.722	M	Balkans17	7950 - 9950
571	Falkenstein	Mathieson Nature 2018	9200	Swabian Jura, Baden-Wurttemberg, Falkenstein-Höhle	Germany	48.06	9.04	M	C_Europe17	7950 - 9950
6546	I3025	Brace Diekmann Nature Ecology Evolution 2019	9130	England, Devon, Torquay, Kent's Cavern	Great Britain	50.468169	-3.503071	F	Britain17	7950 - 9950
6464	R15	Antonio Gao Moots Science 2019	9125	Grotta Continenza	Italy	41.96	13.54	M	Apennine17	7950 - 9950
4129	I1875	Mathieson Nature 2018	9118	Vela Spila	Croatia	42.96723	16.70719	F	Balkans17	7950 - 9950
6640	I5436	Mathieson Nature 2018	9025	Schela Cladovei	Romania	44.629711	22.612556	F	Balkans17	7950 - 9950
4915	I2966	Skoglund Cell 2017	8955	Hora	Malawi	-11.65993	33.641921	M	S_Africa17	7950 - 9950
4590	I4607	Mathieson Nature 2018	8940	Schela Cladovei	Romania	44.629711	22.612556	M	Balkans17	7950 - 9950
4386	I5401	Mathieson Nature 2018	8838	Hajduka Vodenica	Serbia	44.640262	22.30333	M	Balkans17	7950 - 9950
4478	M96	Gonzales Fortes Current Biology 2017	8825	Schela Cladovei	Romania	44.629711	22.612556	M	Balkans17	7950 - 9950
4477	M95	Gonzales Fortes Current Biology 2017	8814	Schela Cladovei	Romania	44.629711	22.612556	M	Balkans17	7950 - 9950
1524	I4655	Mathieson Nature 2018	8765	Schela Cladovei	Romania	44.629711	22.612556	M	Balkans17	7950 - 9950
4155	I4916	Mathieson Nature 2018	8763	Hajduka Vodenica	Serbia	44.640262	22.30333	M	Balkans17	7950 - 9950
4599	I4870	Mathieson Nature 2018	8740	Vlasac	Serbia	44.53368	22.05032	M	Balkans17	7950 - 9950
4479	I5408	Gonzales Fortes Current Biology 2017	8704	Ostrovul Corbului	Romania	44.517	22.722	M	Balkans17	7950 - 9950
4585	I4582	Mathieson Nature 2018	8697	Ostrovul Corbului	Romania	44.517	22.722	F	Balkans17	7950 - 9950

4392	I5411	Mathieson Nature 2018	8600	Schela Cladovei	Romania	44.629711	22.612556	M	Balkans17	7950 - 9950
4144	I4874	Mathieson Nature 2018	8506	Vlasac	Serbia	44.53368	22.05032	F	Balkans17	7950 - 9950
4147	I4877	Mathieson Nature 2018	8505	Vlasac	Serbia	44.53368	22.05032	F	Balkans17	7950 - 9950
4145	I4875	Mathieson Nature 2018	8458	Vlasac	Serbia	44.53368	22.05032	F	Balkans17	7950 - 9950
4146	I4876	Mathieson Nature 2018	8455	Vlasac	Serbia	44.53368	22.05032	F	Balkans17	7950 - 9950
4141	I4871	Mathieson Nature 2018	8450	Vlasac	Serbia	44.53368	22.05032	F	Balkans17	7950 - 9950
4142	I4872	Mathieson Nature 2018	8450	Vlasac	Serbia	44.53368	22.05032	F	Balkans17	7950 - 9950
4243	I5772	Mathieson Nature 2018	8450	Vlasac	Serbia	44.53368	22.05032	M	Balkans17	7950 - 9950
4150	I4881	Mathieson Nature 2018	8363	Vlasac	Serbia	44.53368	22.05032	M	Balkans17	7950 - 9950
4242	I5771	Mathieson Nature 2018	8325	Vlasac	Serbia	44.53368	22.05032	M	Balkans17	7950 - 9950
4387	I5402	Mathieson Nature 2018	8156	Hajduka Vodenica	Serbia	44.640262	22.30333	M	Balkans17	7950 - 9950
4153	I4914	Mathieson Nature 2018	8123	Hajduka Vodenica	Serbia	44.640262	22.30333	M	Balkans17	7950 - 9950
4154	I4915	Mathieson Nature 2018	8115	Hajduka Vodenica	Serbia	44.640262	22.30333	M	Balkans17	7950 - 9950
5067	I2967	Skoglund Cell 2017	8065	Hora	Malawi	-11.65993	33.641921	F	S_Africa17	7950 - 9950
4156	I4917	Mathieson Nature 2018	8058	Hajduka Vodenica	Serbia	44.640262	22.30333	F	Balkans17	7950 - 9950
478	I0001	Lazaridis Nature 2014	8050	Echternach	Luxembourg	49.81	6.4	M	C_Europe17	7950 - 9950
4176	I5233	Mathieson Nature 2018	8001	Padina	Serbia	44.595879	22.010568	F	Balkans17	7950 - 9950
4151	I4882	Mathieson Nature 2018	8000	Vlasac	Serbia	44.53368	22.05032	M	Balkans17	7950 - 9950
4175	I5232	Mathieson Nature 2018	7901	Padina	Serbia	44.595879	22.010568	M	Balkans18	5950 - 7950
5934	I10871	Lipson Nature 2020	7890	Shum Laka	Cameroon	5.8586	10.0778	M	W_Africa18	5950 - 7950

4143	I4873	Mathieson Nature 2018	7872	Vlasac	Serbia	44.53368	22.05032	F	Balkans18	5950 - 7950
660	I0843	Olalde Science 2019	7853	Leon, La Brana-Arintero	Spain	42.911	-5.3778	M	Iberia18	5950 - 7950
4464	I3209	Olalde Science 2019	7830	Castellón, Ares del Maestre, Cingle Mas Nou,	Spain	40.418196	-0.11675	M	Iberia18	5950 - 7950
661	I0585	Mathieson Nature 2015	7815	Leon, La Brana-Arintero	Spain	42.911	-5.3778	M	Iberia18	5950 - 7950
902	I0585	Olalde Nature 2014	7815	Leon, La Brana-Arintero	Spain	42.911	-5.3778	M	Iberia18	5950 - 7950
4149	I4880	Mathieson Nature 2018	7813	Vlasac	Serbia	44.53368	22.05032	M	Balkans18	5950 - 7950
4148	I4878	Mathieson Nature 2018	7803	Vlasac	Serbia	44.53368	22.05032	M	Balkans18	5950 - 7950
1384	I2534	Mathieson Nature 2018	7670	Magura Buduiasca, Teleor 3	Romania	44.027196	25.392494	F	Balkans18	5950 - 7950

Figures

Fig. S1 – Unsupervised ADMIXTURE model-based clustering analysis of 77 present-day humans from the Caucasus for K = 2 to 7 clusters. Abbreviations as in **Table 1**.

Fig. S2 – Principal component analysis (PCA) of analyzed human individuals based on genome-wide SNP data. Eigenvectors of principal components are inferred with the modern populations from the Caucasus, while the ancient populations are projected onto these principal components. Bottom left: data points of present-day individuals from the Caucasus plotted with the first 2 PCA axes; Top left: the same data, zoomed on to the populations that lay south of the main ridge of the Greater Caucasus; Bottom right: ancient genomes (centroids of the populations from an indicated geographic area and time frame) projected on the modern Caucasians (background of gray shapes). Abbreviations as in **Table 1**.

Fig. S3 – Genetic affinity (f3) between modern Caucasians and ancient populations. Thick and thin error bars correspond to 1 and 1.96 standard errors of the f3-statistic, respectively.

Fig. S4 – Genetic affinity (f3) between modern populations of the Caucasus and ancient populations (labeled in different colors), plotted against least-cost distance and mean years before present (BP) between the modern and ancient populations.

Fig. S5 – Relationship of f3 statistics between modern populations of the Caucasus and ancient populations (labeled in different colors), taken from different periods of the past time, with the least-cost distance (LCD) between these modern and ancient populations f3 and LCD values are on vertical and horizontal axes, respectively. BP stands for years before present; R^2 is the goodness of fit calculated separately for individual ancient populations and for all ancient populations from a given time period. Regression lines are in corresponding colors, with the overall regression line in black.

Fig. S6 – Genetic affinity (f3) and least-cost paths between modern populations of the Caucasus (labeled in the main maps, see **Table 1** for details) and each of the ancient populations (identified under the inset maps, see **Table 2** for details). Larger points indicate more genetic affinity. The least-cost paths imply that (1) human movement is impeded by terrain ruggedness (TRI), (2) the Bosporus-Dardanelles and the English Channel do not act as barriers, (3) swamps, glaciers and desert are not full barriers, but permeable at the highest of the cost grid values, and (4) riversides in desert are permeable at TRI values. It is assumed that most human movements occurred during climate warming events when the earth's surface was not dramatically different from that of today, and hence the current data of the geographic features are used in the calculation of the least-cost paths. The map is generated using QGIS Desktop 3.10.6-A Coruña (https://qgis.org).

Fig. S7 – Scatter plot and regression line of pairwise Fst and least-cost distance (LCD) between the modern populations of the Caucasus.

Fig. S8 – Neighbor-Joining trees of genetic and geographic distances and ancient ancestry between the modern population hubs in the Caucasus, based on the (1) Fst genetic distances calculated from genome-wide SNP data, (2) Least-cost distances derived by weighting geographic distance with rugged terrain, swamps, glaciers and desert, and (3) difference in ancient genetic ancestry (f3-statistic). See **Table 1** for population abbreviations.

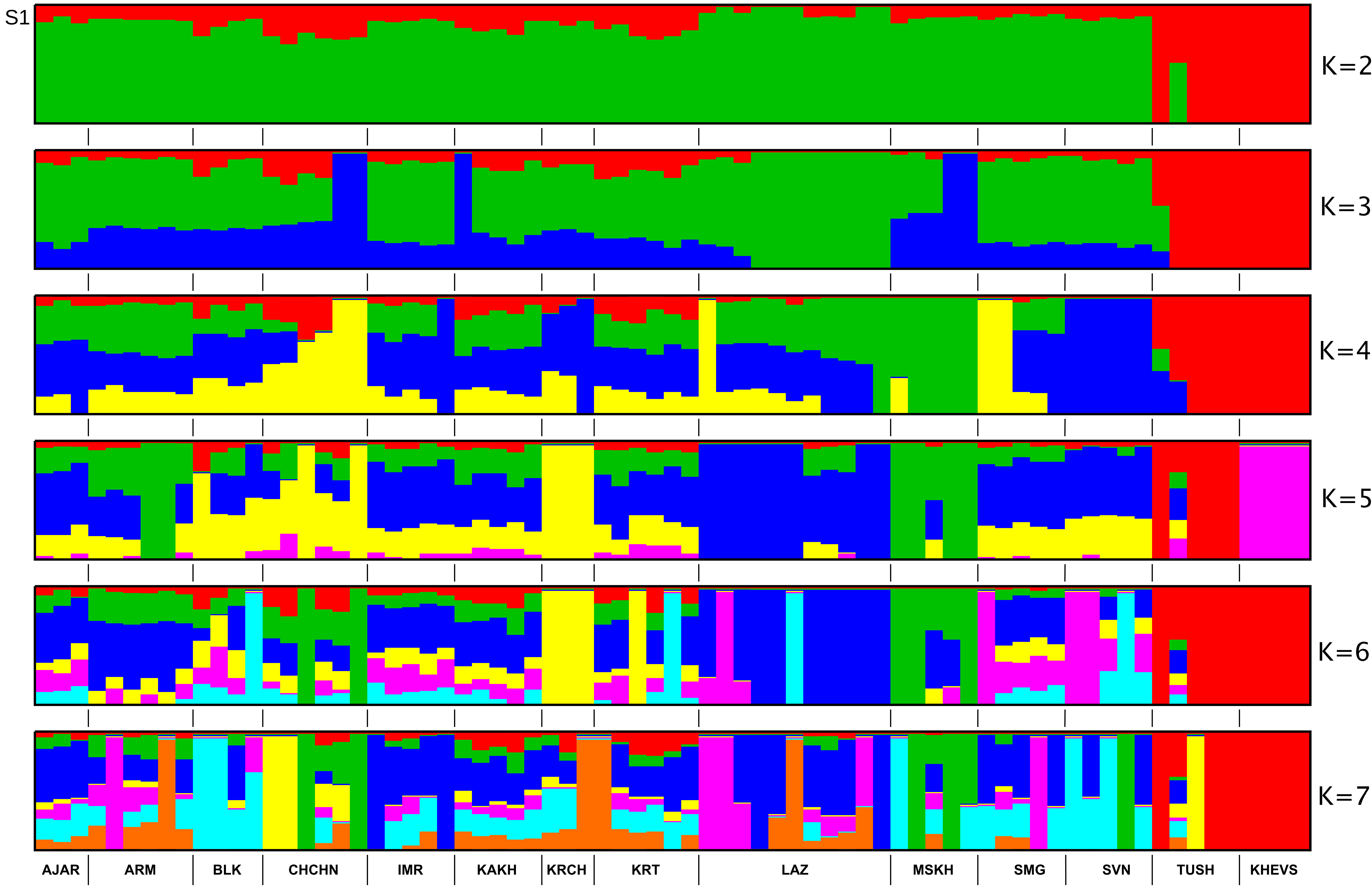
S1
K=2
K=3
K=4
K=5
K=6
K=7
AJAR
ARM
BLK
CHCHN
IMR
KAKH
KRCH
KRT
LAZ
MSKH
SMG
SVN
TUSH
KHEVS

S2

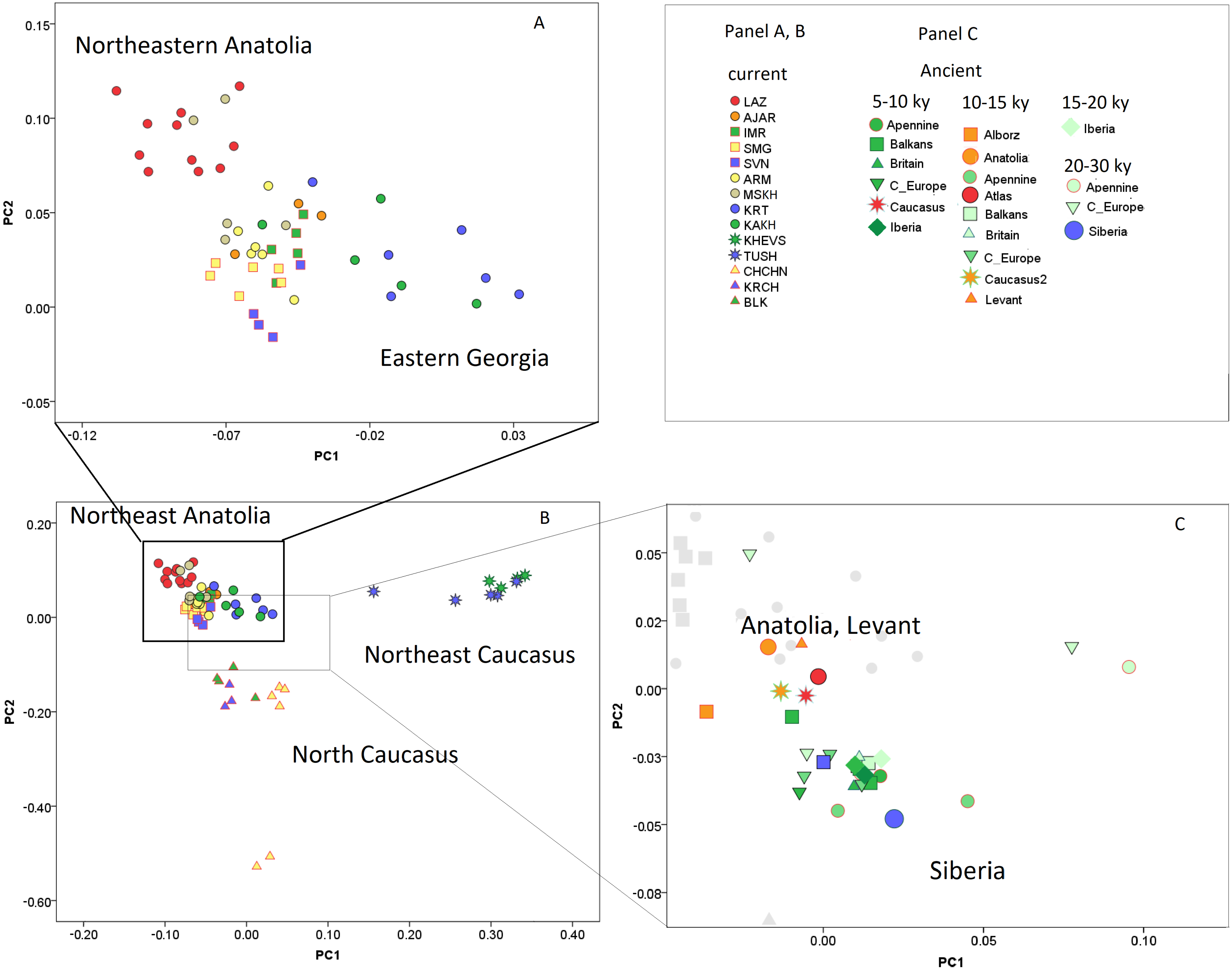
A
Northeastern Anatolia
Eastern Georgia
PC1
PC2
Panel A, B
current
LAZ
AJAR
IMR
SMG
SVN
ARM
MSKH
KRT
KAKH
KHEVS
TUSH
CHCHN
KRCH
BLK
Panel C
Ancient
5-10 ky
Apennine
Balkans
Britain
C_Europe
Caucasus
Iberia
10-15 ky
Alborz
Anatolia
Apennine
Atlas
Balkans
Britain
C_Europe
Caucasus2
Levant
15-20 ky
Iberia
20-30 ky
Apennine
C_Europe
Siberia
B
Northeast Anatolia
Northeast Caucasus
North Caucasus
C
Anatolia, Levant
Siberia

S3

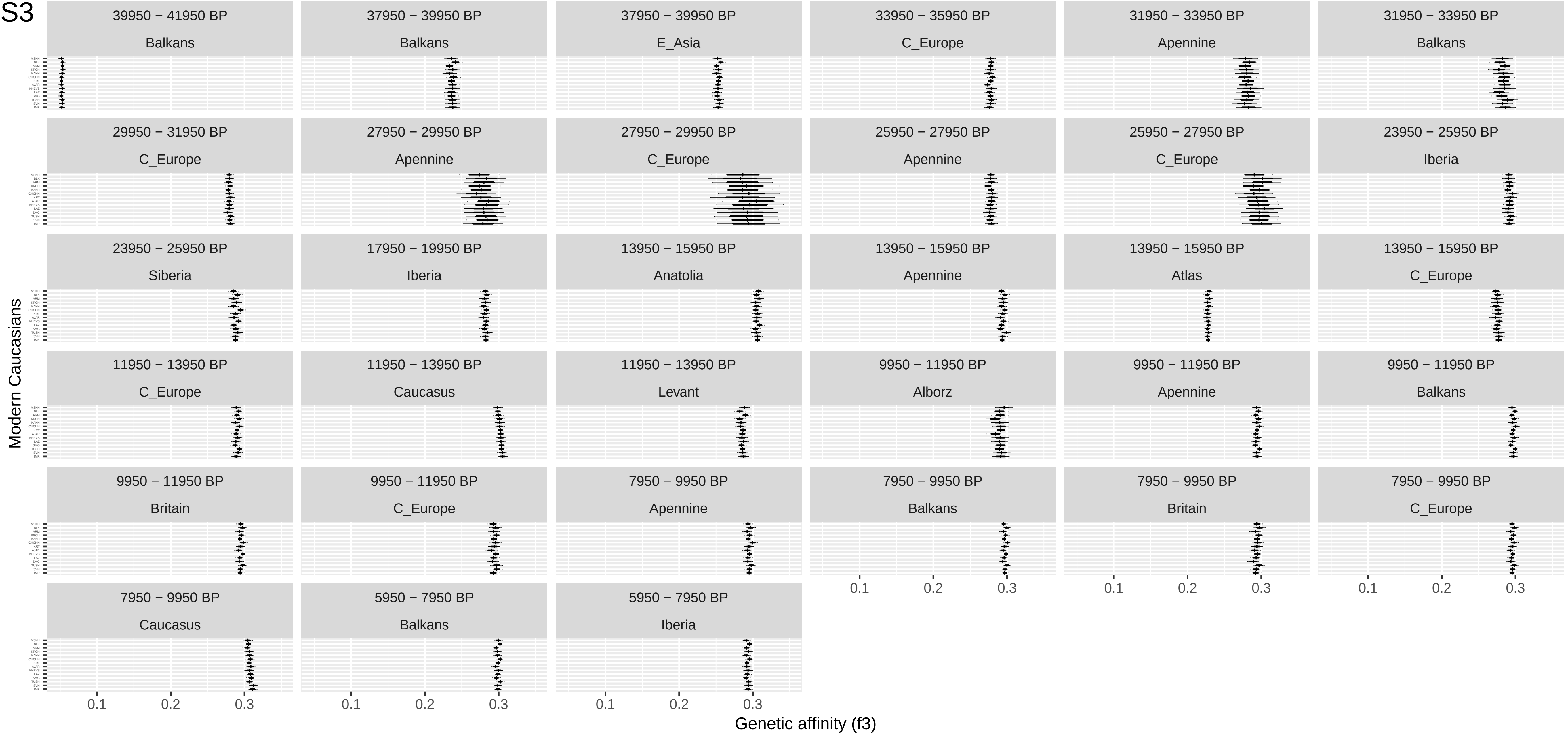
39950 − 41950 BP
Balkans
37950 − 39950 BP
Balkans
37950 − 39950 BP
E_Asia
33950 − 35950 BP
C_Europe
31950 − 33950 BP
Apennine
31950 − 33950 BP
Balkans
29950 − 31950 BP
C_Europe
27950 − 29950 BP
Apennine
27950 − 29950 BP
C_Europe
25950 − 27950 BP
Apennine
25950 − 27950 BP
C_Europe
23950 − 25950 BP
Iberia
23950 − 25950 BP
Siberia
17950 − 19950 BP
Iberia
13950 − 15950 BP
Anatolia
13950 − 15950 BP
Apennine
13950 − 15950 BP
Atlas
13950 − 15950 BP
C_Europe
11950 − 13950 BP
C_Europe
11950 − 13950 BP
Caucasus
11950 − 13950 BP
Levant
9950 − 11950 BP
Alborz
9950 − 11950 BP
Apennine
9950 − 11950 BP
Balkans
9950 − 11950 BP
Britain
9950 − 11950 BP
C_Europe
7950 − 9950 BP
Apennine
7950 − 9950 BP
Balkans
7950 − 9950 BP
Britain
7950 − 9950 BP
C_Europe
7950 − 9950 BP
Caucasus
5950 − 7950 BP
Balkans
5950 − 7950 BP
Iberia
0.1
0.2
0.3
Modern Caucasians
Genetic affinity (f3)

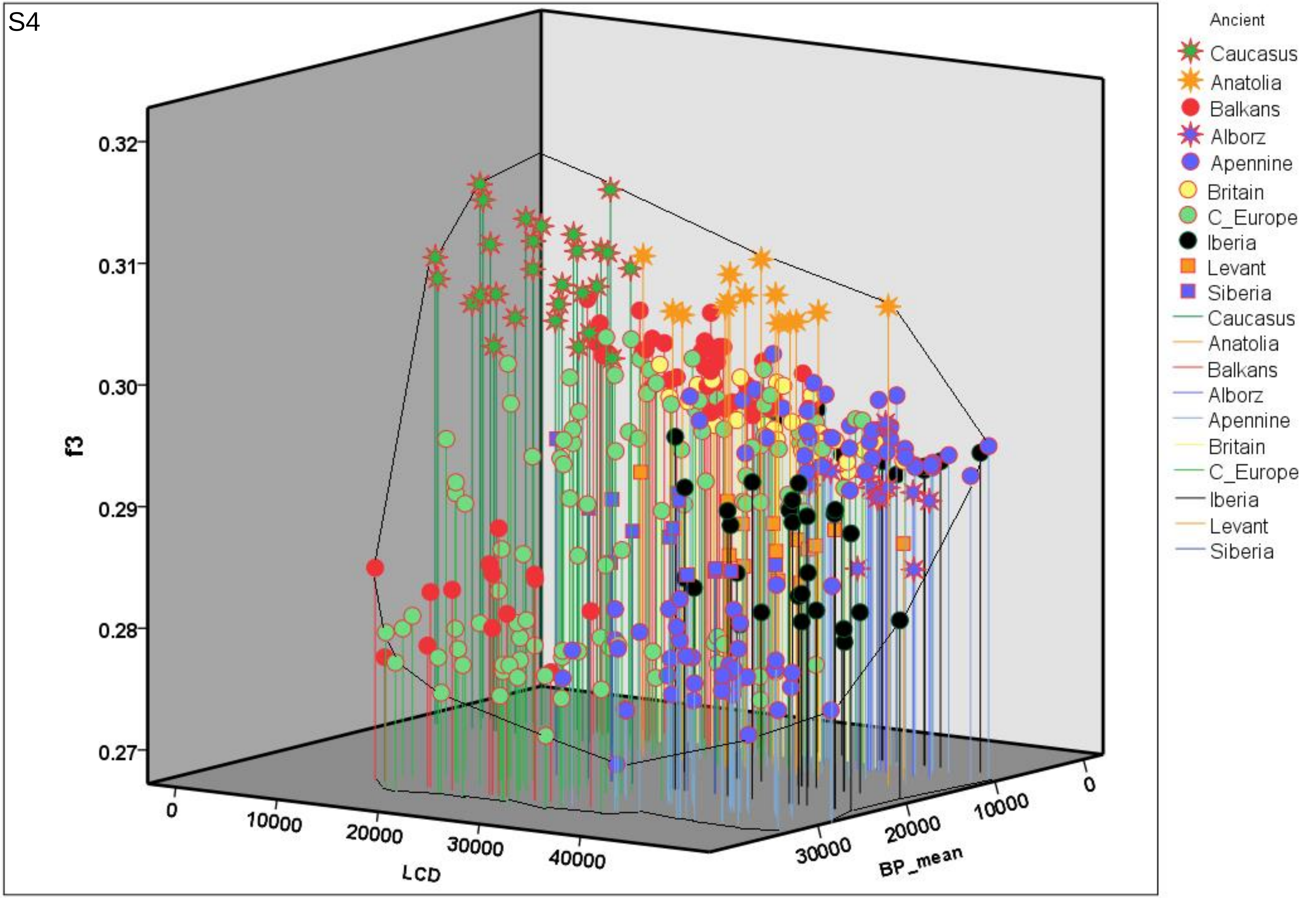
S4
f3
0.32
0.31
0.30
0.29
0.28
0.27
0
10000
20000
30000
40000
LCD
0
10000
20000
30000
BP_mean
Ancient
Caucasus
Anatolia
Balkans
Alborz
Apennine
Britain
C_Europe
Iberia
Levant
Siberia
Caucasus
Anatolia
Balkans
Alborz
Apennine
Britain
C_Europe
Iberia
Levant
Siberia

S5

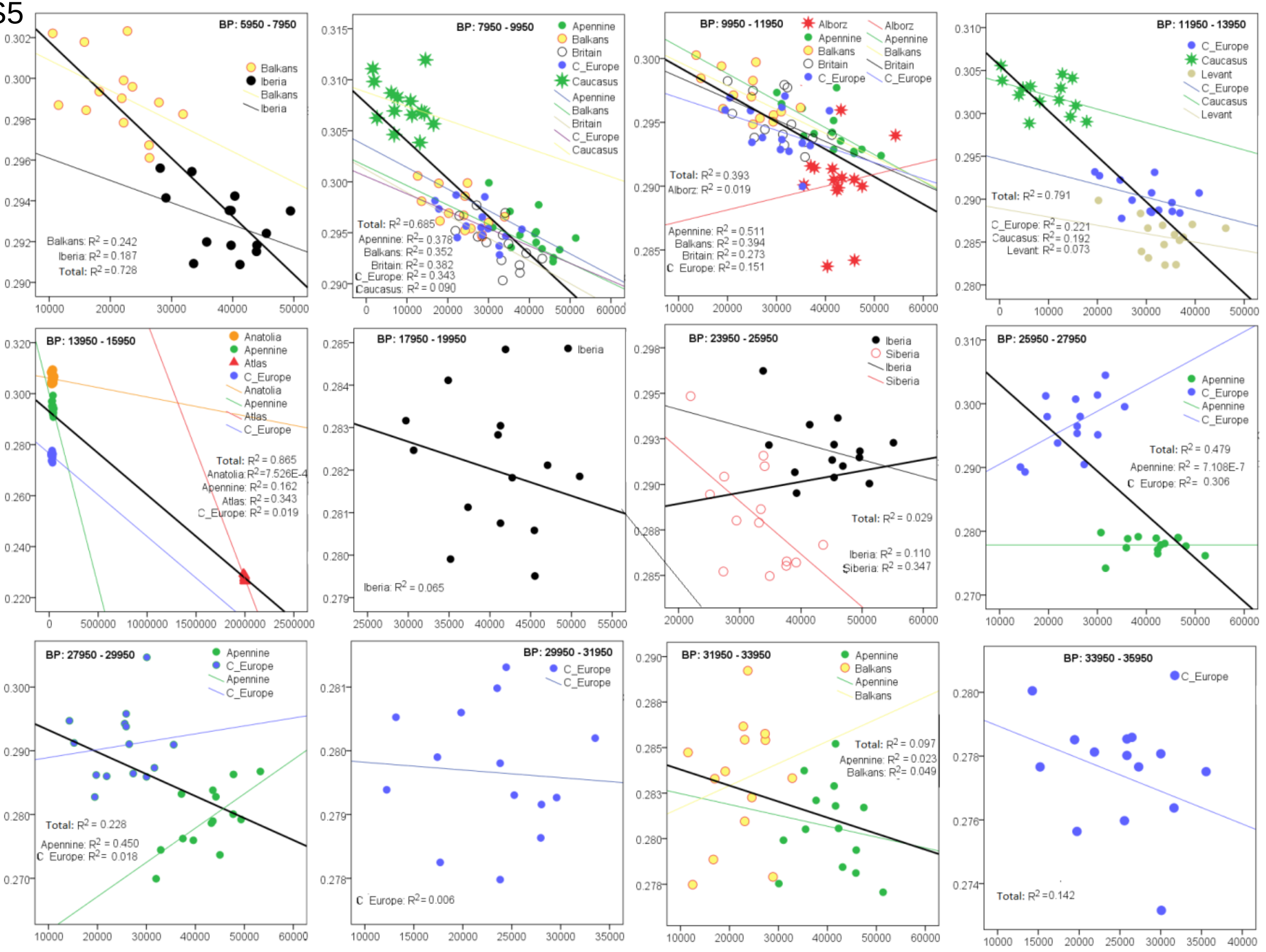

BP: 5950 - 7950
Balkans
Iberia
Balkans: R^2 = 0.242
Iberia: R^2 = 0.187
Total: R^2 = 0.728
BP: 7950 - 9950
Apennine
Balkans
Britain
C_Europe
Caucasus
Total: R^2 = 0.685
Apennine: R^2 = 0.378
Balkans: R^2 = 0.352
Britain: R^2 = 0.382
C_Europe: R^2 = 0.343
Caucasus: R^2 = 0.090
BP: 9950 - 11950
Alborz
Apennine
Balkans
Britain
C_Europe
Total: R^2 = 0.393
Alborz: R^2 = 0.019
Apennine: R^2 = 0.511
Balkans: R^2 = 0.394
Britain: R^2 = 0.273
C_Europe: R^2 = 0.151
BP: 11950 - 13950
C_Europe
Caucasus
Levant
Total: R^2 = 0.791
C_Europe: R^2 = 0.221
Caucasus: R^2 = 0.192
Levant: R^2 = 0.073
BP: 13950 - 15950
Anatolia
Apennine
Atlas
C_Europe
Total: R^2 = 0.865
Anatolia: R^2 = 7.526E-4
Apennine: R^2 = 0.162
Atlas: R^2 = 0.343
C_Europe: R^2 = 0.019
BP: 17950 - 19950
Iberia
Iberia: R^2 = 0.065
BP: 23950 - 25950
Iberia
Siberia
Total: R^2 = 0.029
Iberia: R^2 = 0.110
Siberia: R^2 = 0.347
BP: 25950 - 27950
Apennine
C_Europe
Total: R^2 = 0.479
Apennine: R^2 = 7.108E-7
C_Europe: R^2 = 0.306
BP: 27950 - 29950
Apennine
C_Europe
Total: R^2 = 0.228
Apennine: R^2 = 0.450
C_Europe: R^2 = 0.018
BP: 29950 - 31950
C_Europe
C_Europe: R^2 = 0.006
BP: 31950 - 33950
Apennine
Balkans
Total: R^2 = 0.097
Apennine: R^2 = 0.023
Balkans: R^2 = 0.049
BP: 33950 - 35950
C_Europe
Total: R^2 = 0.142

S6

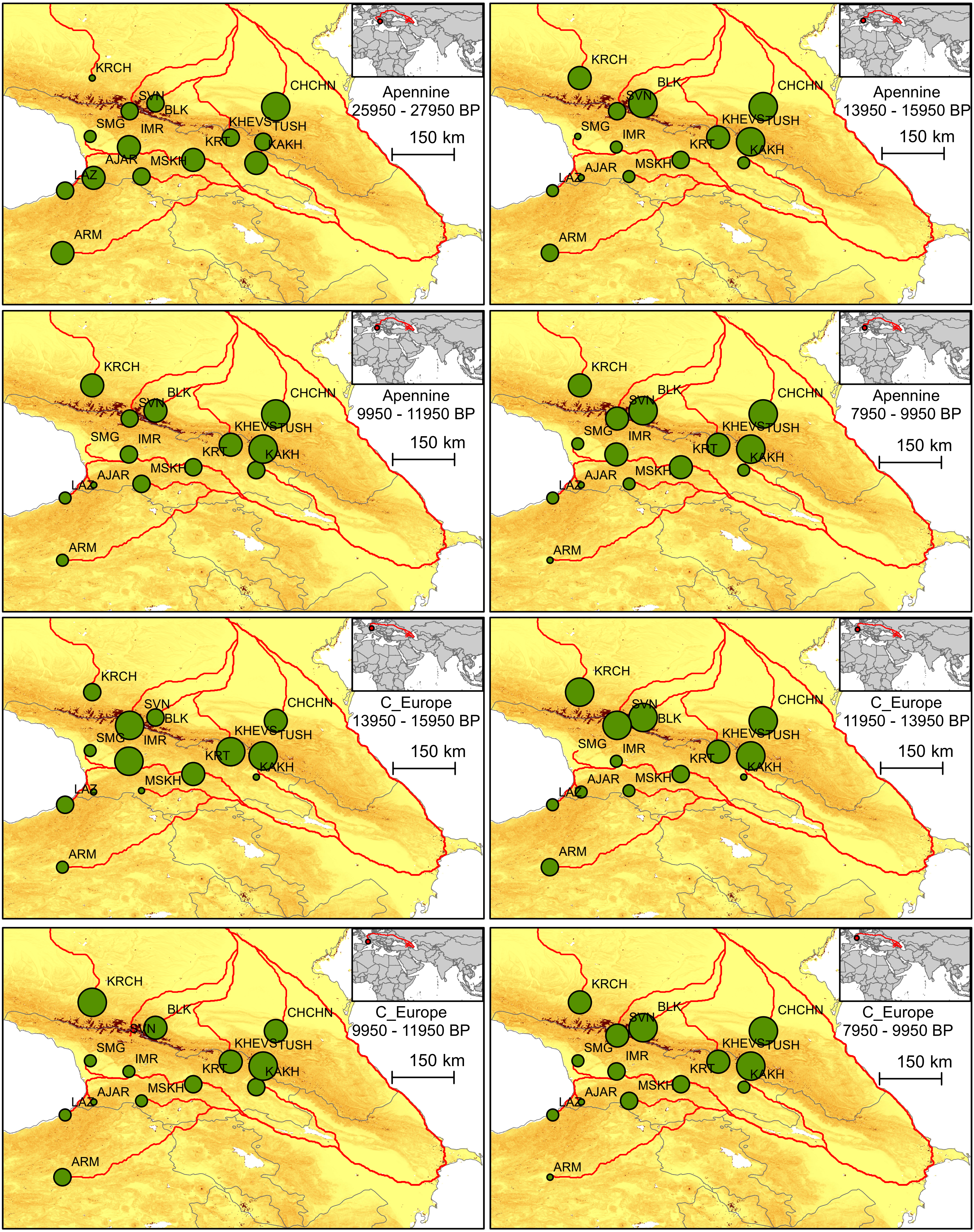
KRCH
SVN
BLK
CHCHN
SMG
IMR
KHEVS
TUSH
KRT
KAKH
AJAR
MSKH
LAZ
ARM
Apennine
25950 - 27950 BP
150 km
KRCH
BLK
SVN
CHCHN
SMG
IMR
KHEVS
TUSH
KRT
KAKH
AJAR
MSKH
LAZ
ARM
Apennine
13950 - 15950 BP
150 km
KRCH
BLK
SVN
CHCHN
SMG
IMR
KHEVS
TUSH
KRT
KAKH
AJAR
MSKH
LAZ
ARM
Apennine
9950 - 11950 BP
150 km
KRCH
BLK
SVN
CHCHN
SMG
IMR
KHEVS
TUSH
KRT
KAKH
AJAR
MSKH
LAZ
ARM
Apennine
7950 - 9950 BP
150 km
KRCH
SVN
BLK
CHCHN
SMG
IMR
KHEVS
TUSH
KRT
KAKH
MSKH
LAZ
ARM
C_Europe
13950 - 15950 BP
150 km
KRCH
SVN
BLK
CHCHN
SMG
IMR
KHEVS
TUSH
KRT
KAKH
AJAR
MSKH
LAZ
ARM
C_Europe
11950 - 13950 BP
150 km
KRCH
BLK
SVN
CHCHN
SMG
IMR
KHEVS
TUSH
KRT
KAKH
AJAR
MSKH
LAZ
ARM
C_Europe
9950 - 11950 BP
150 km
KRCH
BLK
SVN
CHCHN
SMG
IMR
KHEVS
TUSH
KRT
KAKH
AJAR
MSKH
LAZ
ARM
C_Europe
7950 - 9950 BP
150 km

S6

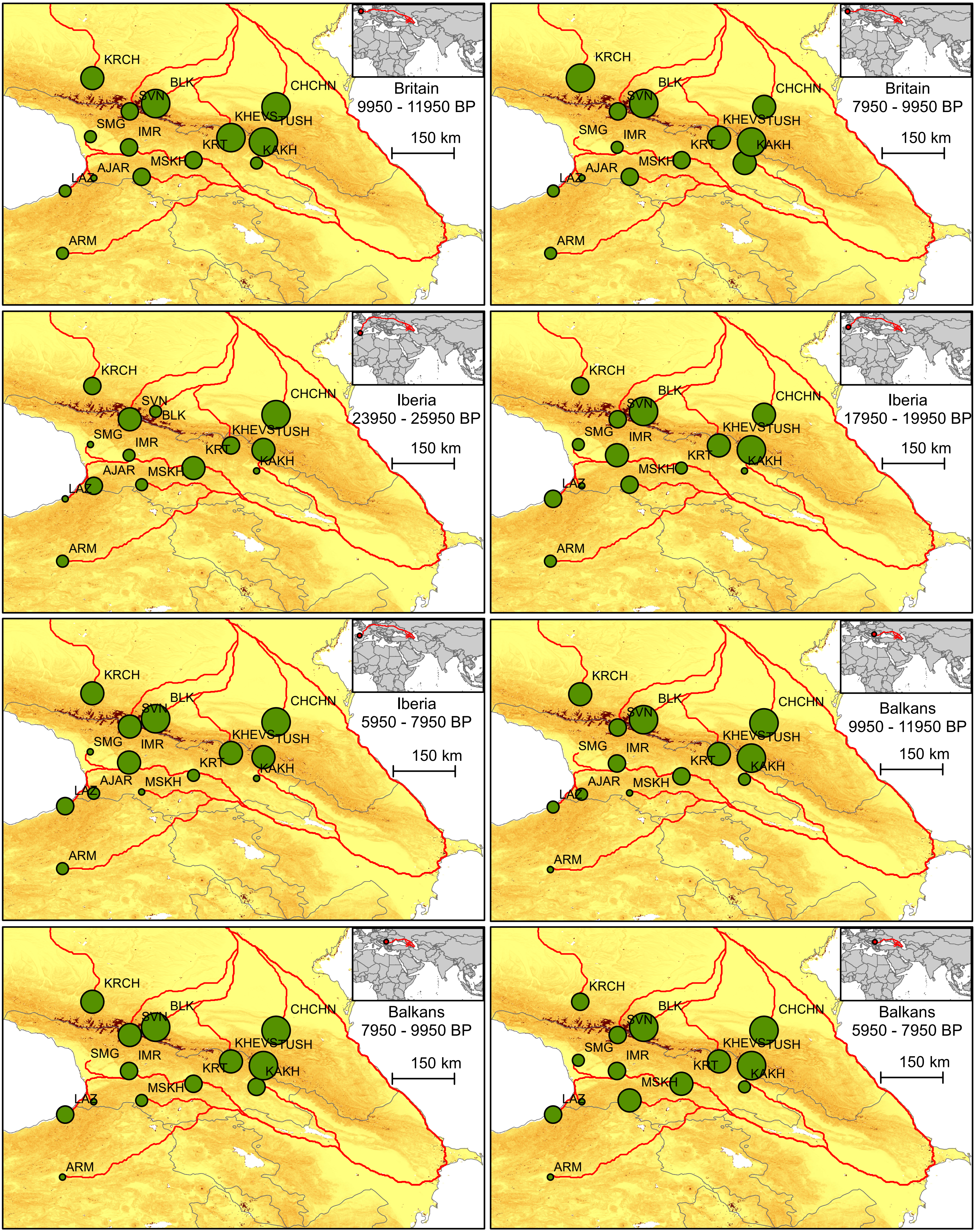
KRCH
BLK
SVN
CHCHN
KHEVS
TUSH
SMG
IMR
KRT
KAKH
MSKH
AJAR
LAZ
ARM
Britain
9950 - 11950 BP
150 km
Britain
7950 - 9950 BP
150 km
Iberia
23950 - 25950 BP
150 km
Iberia
17950 - 19950 BP
150 km
Iberia
5950 - 7950 BP
150 km
Balkans
9950 - 11950 BP
150 km
Balkans
7950 - 9950 BP
150 km
Balkans
5950 - 7950 BP
150 km

S6

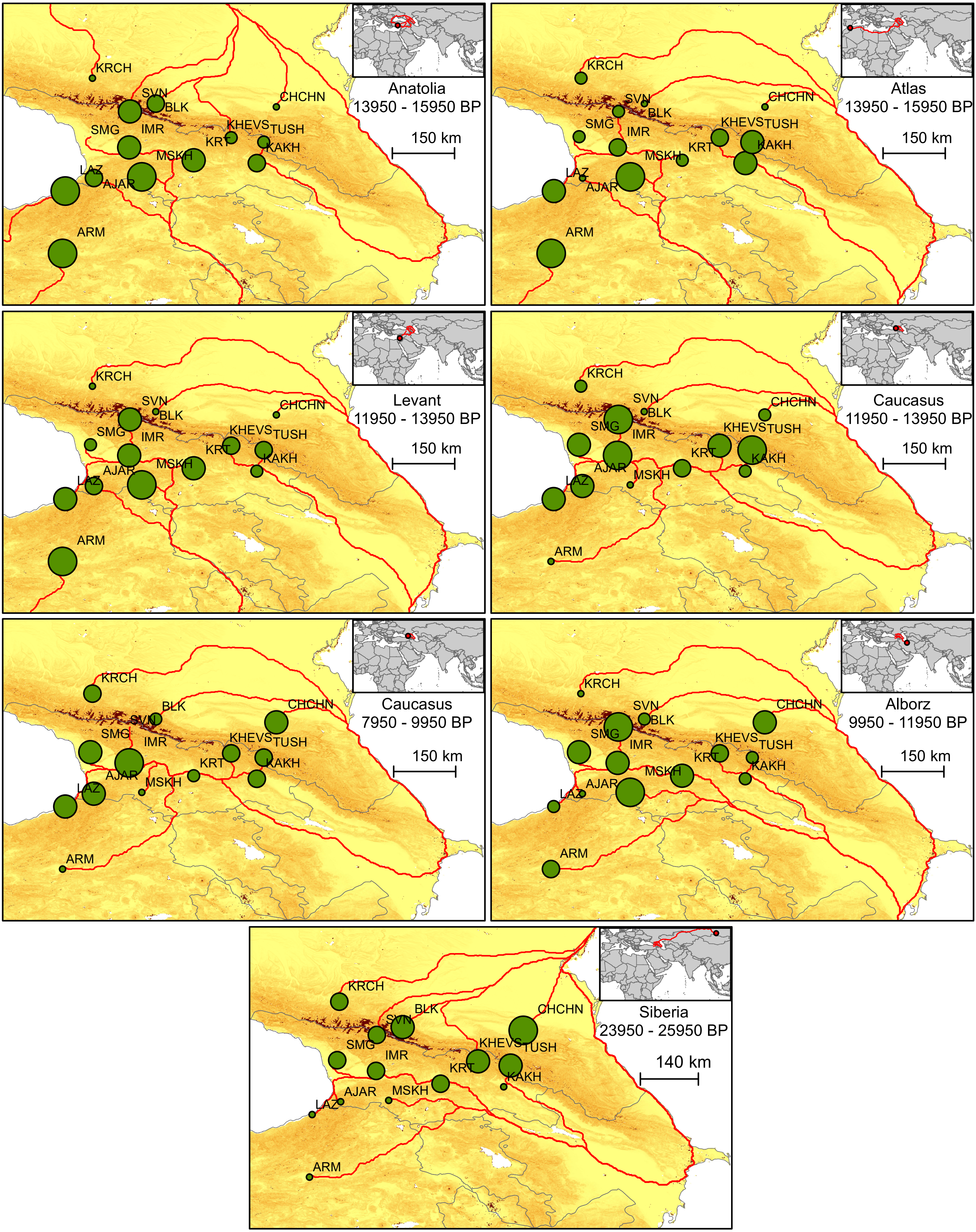
Anatolia
13950 - 15950 BP
150 km
KRCH
SVN
BLK
CHCHN
SMG
IMR
KHEVS
TUSH
KRT
KAKH
MSKH
LAZ
AJAR
ARM
Atlas
13950 - 15950 BP
150 km
Levant
11950 - 13950 BP
150 km
Caucasus
11950 - 13950 BP
150 km
Caucasus
7950 - 9950 BP
150 km
Alborz
9950 - 11950 BP
150 km
Siberia
23950 - 25950 BP
140 km

S7

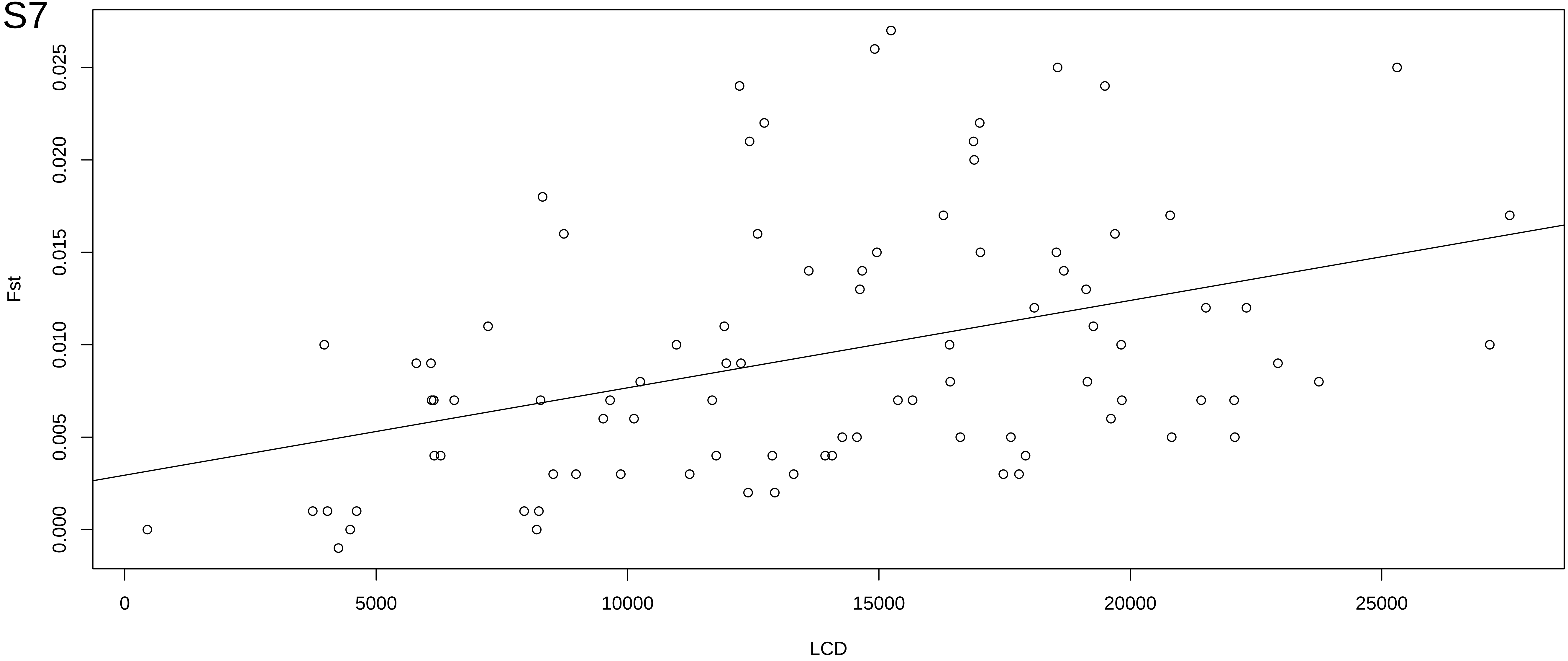

Fst
0.000
0.005
0.010
0.015
0.020
0.025
0
5000
10000
15000
20000
25000
LCD

S8

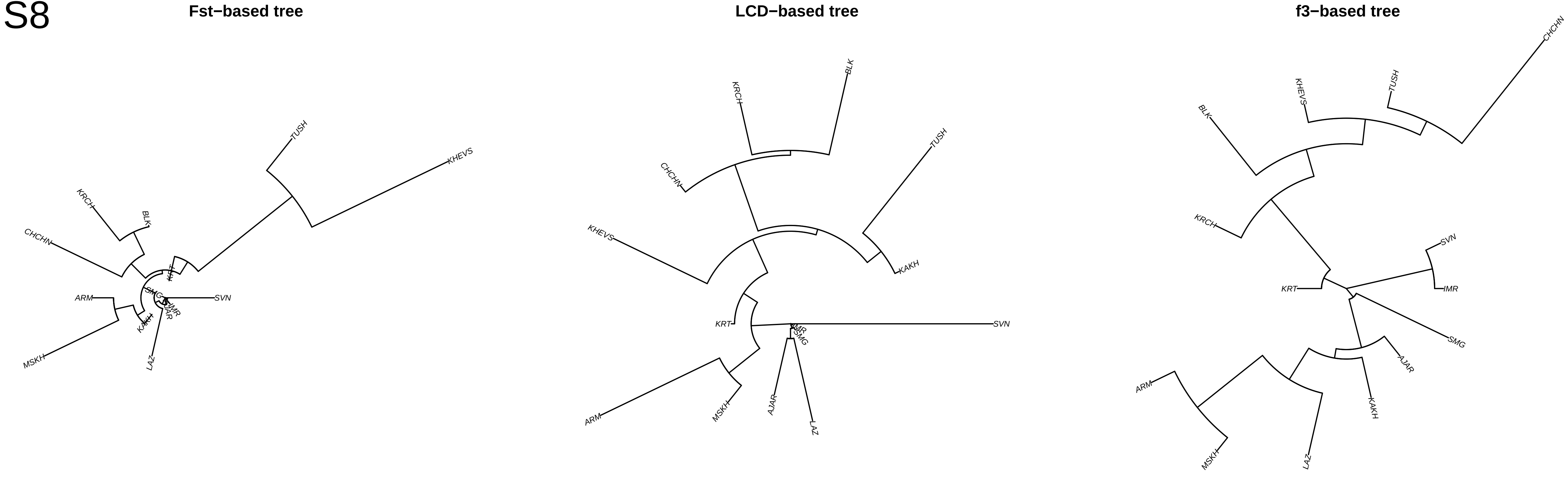
Fst−based tree
TUSH
KHEVS
KRCH
BLK
CHCHN
KRT
SMG
ARM
SVN
IMR
AJAR
KAKH
MSKH
LAZ
LCD−based tree
BLK
KRCH
TUSH
CHCHN
KHEVS
KAKH
KRT
SVN
IMR
SMG
ARM
MSKH
AJAR
LAZ
f3−based tree
CHCHN
TUSH
KHEVS
BLK
KRCH
SVN
KRT
IMR
SMG
AJAR
ARM
KAKH
MSKH
LAZ
